# Supplementary material for: CasPlay provides a gRNA-barcoded CRISPR-based display platform for antibody repertoire profiling
Source: Cell Rep Methods. 2022 Oct 17;2(10):100318. doi: 10.1016/j.crmeth.2022.100318 (PMC9606310; doi:10.1016/j.crmeth.2022.100318)
Supplement: Document S1. Figures S1–S6 and Table S1 [file mmc1.pdf]

Cell Reports Methods, Volume *2*

Supplemental information

CasPlay provides a gRNA-barcoded CRISPR-based display platform for antibody repertoire profiling

Karl W. Barber, Ellen Shrock, and Stephen J. Elledge

Supplementary Fig. 1: Detailed CasPlay plasmid library cloning overview, related to STAR Methods

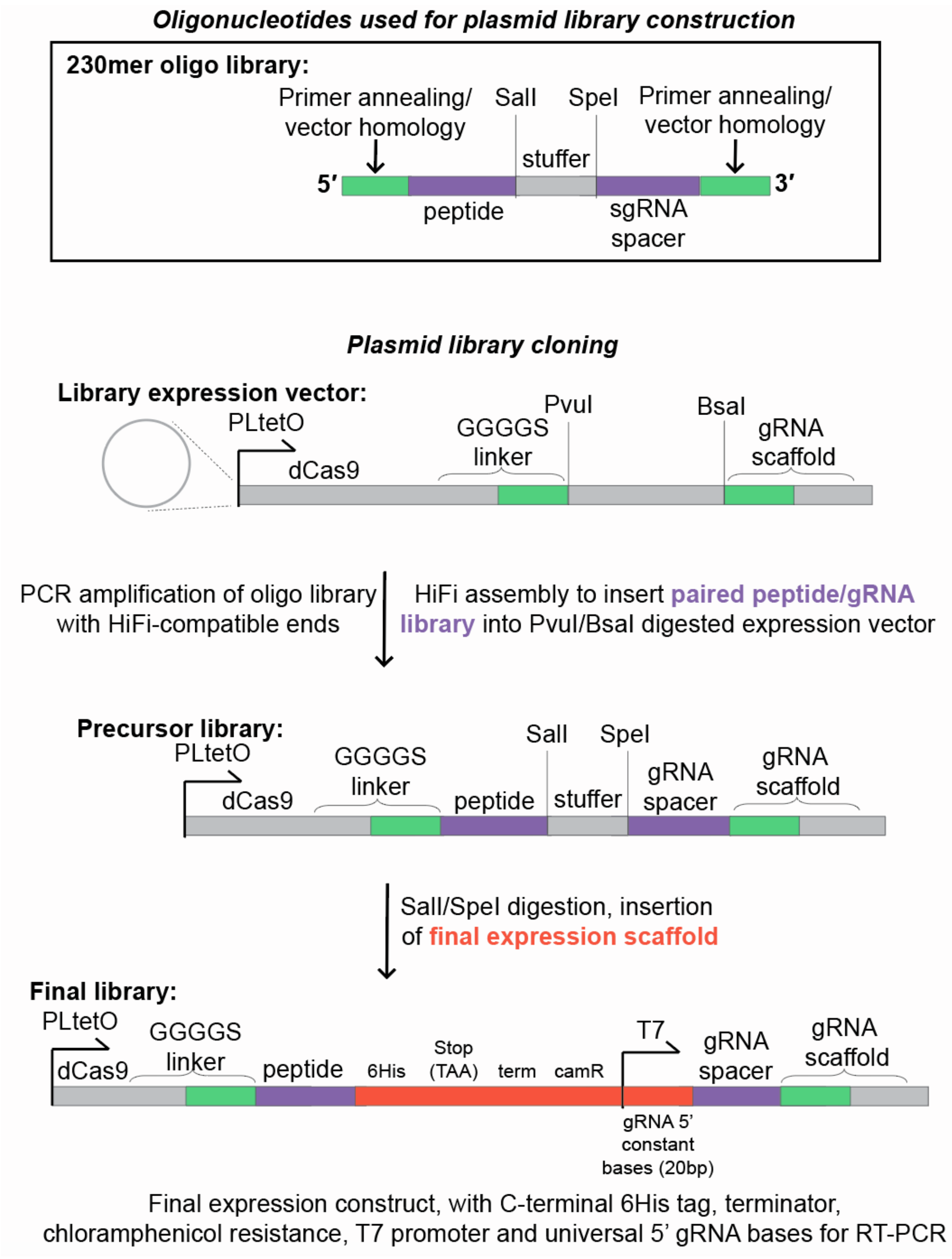

Schematic overview of CasPlay oligonucleotide library DNA sequence features and library cloning methods.

Supplementary Fig. 2: Cross-platform comparison of SARS-CoV-2 epitope mapping, related to Fig. 3

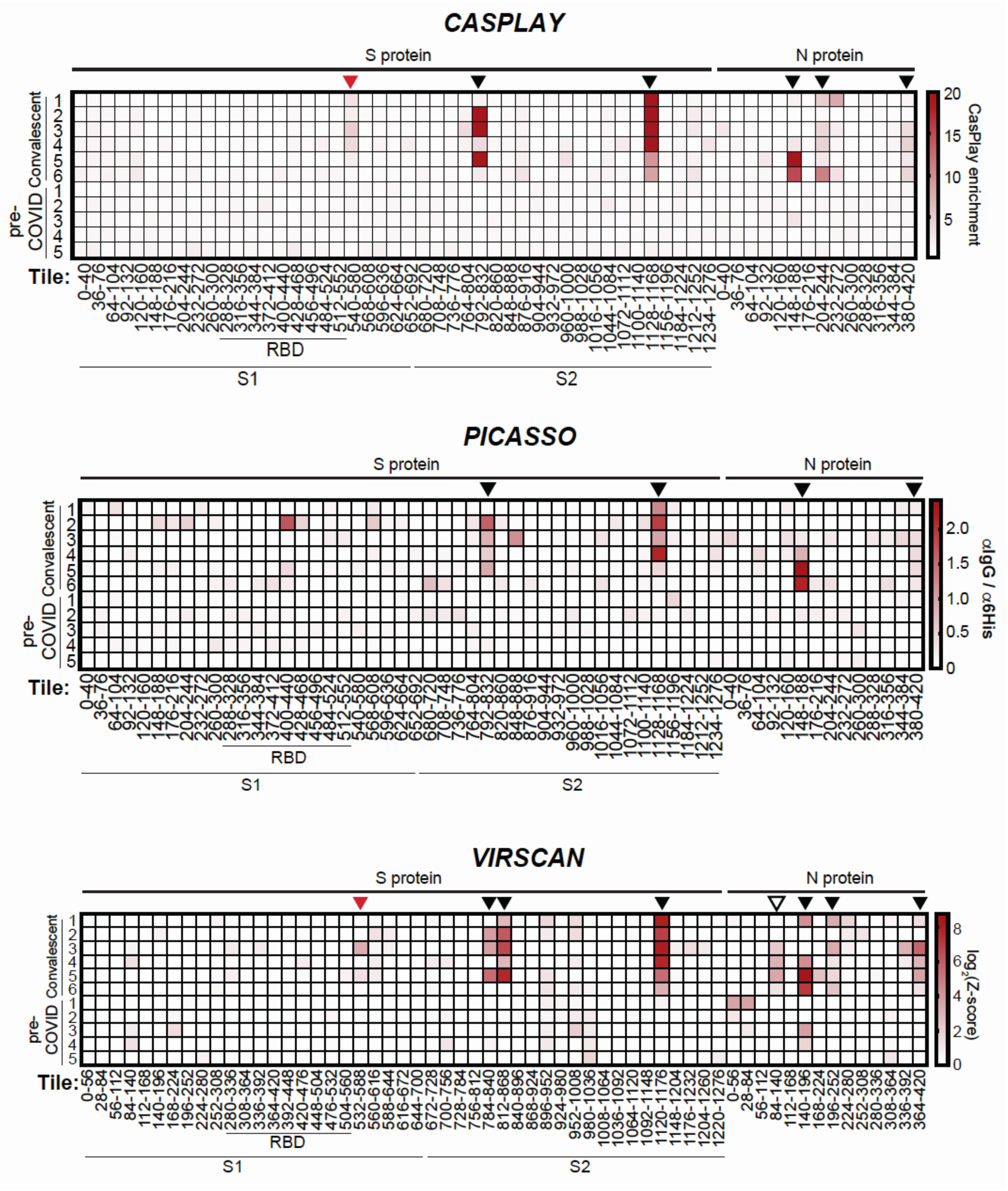

Matched patient sample data (6 convalescent patient serum samples, 5 pre-COVID serum samples) using tiled peptides representing SARS-CoV-2 shown. PICASSO and VirScan data obtained in and adapted from Barber et al., Molecular Cell 2021(Barber et al., 2021). Black arrowheads indicate epitopes recognized by two or more patients in this dataset above signal thresholds (CasPlay enrichment ≥ 3.5, PICASSO signal ratio ≥ 1.3, VirScan z-score ≥ 3.5). White arrowhead indicates epitope identified only by VirScan. Red arrowhead indicates epitope identified in both CasPlay and VirScan but not PICASSO, but only in one patient in this set.

Supplementary Fig. 3: CasPlay human virome library characterization and control experiments, related to Fig. 4

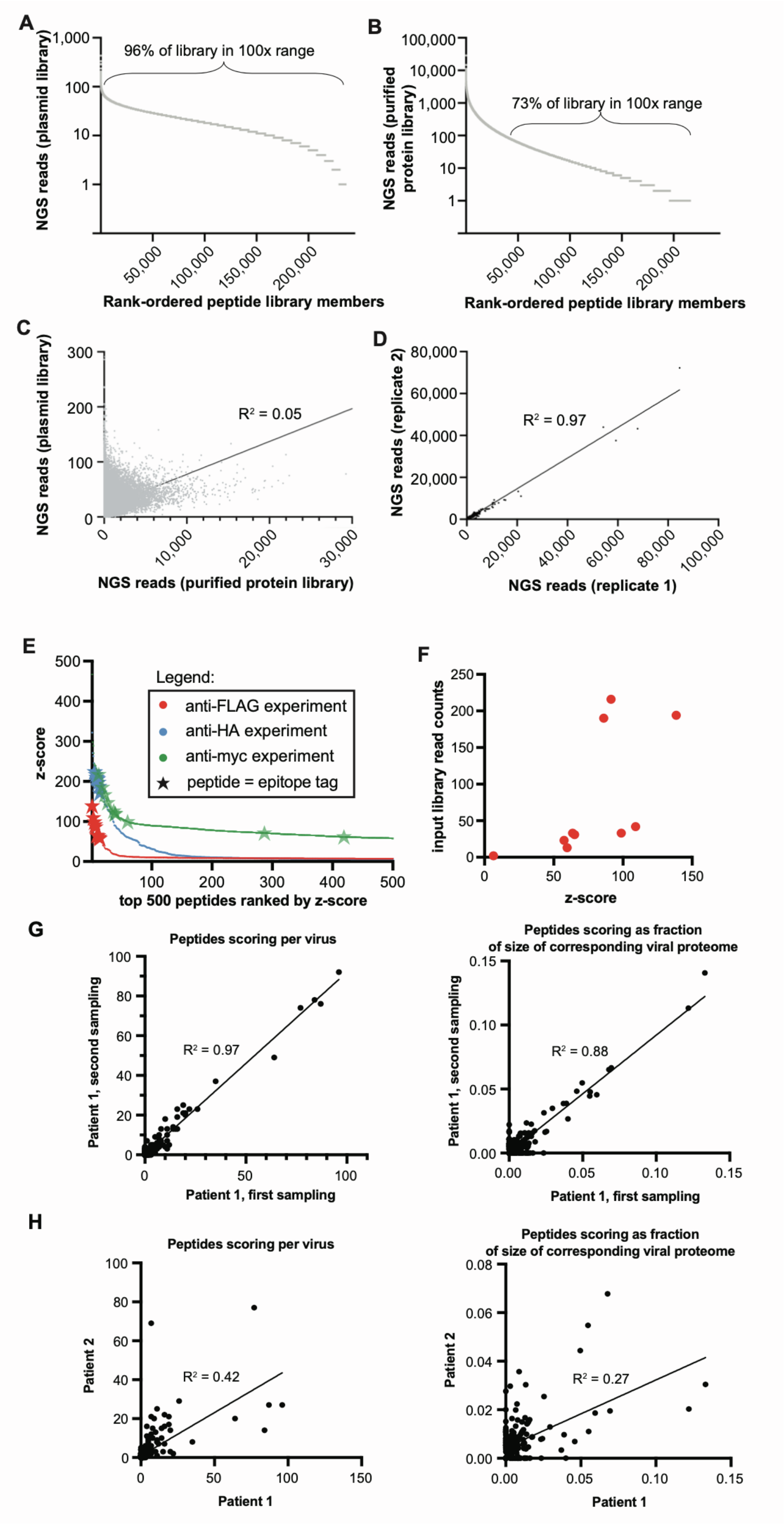

(**A**) Sequencing read distribution of 245,002 barcodes in final plasmid library encoding dCas9-peptide fusion and gRNA pairs. (**B**) Sequencing read distribution of 245,002 barcodes from RT-PCR of gRNAs from final purified dCas9-fusion library preparations. (**C**) No correlation between gRNA barcode read counts in final plasmid library and purified protein library. Three data points did not fall into the plotted range. (**D**) High correlation between independent sample replicates (from two separate transformations of the plasmid library) of gRNA barcodes sequenced from purified protein library. (**E**) Top 500 enriched peptides from the 245,002-member CasPlay library experiments using anti-FLAG, anti-HA or anti-myc antibody ranked by z-score of corresponding gRNA barcode. Epitope tag replicates corresponding to the designated antibody indicated by star symbols. Peptide z-scores averaged for two independent sample replicates. All expected control peptides are plotted, with the exception of one FLAG peptide which ranked #525 in the anti-FLAG immunoprecipitation. (**F**) Comparison of CasPlay z-scores for the FLAG control peptides in the anti-FLAG immunoprecipitation experiment and the barcode read counts in the 245,002-member CasPlay input library, averaged for two independent sample replicates. (**G**) Example of stability of measured antibody repertoire by CasPlay demonstrated by number of peptide hits (z-score ≥ 3.5, left) or peptide hits as a fraction of the size of a given viral proteome (right) in the CasPlay library from two patient-matched longitudinal samples, taken between two weeks and three months apart. (**H**) Example of inconsistency of peptide hits as total peptide hits (z-score ≥ 3.5, left) or peptide hits as fraction of size of corresponding viral proteome (right) for two unrelated samples.

Supplementary Fig. 4: Comparison of CasPlay to VirScan, related to Fig. 4

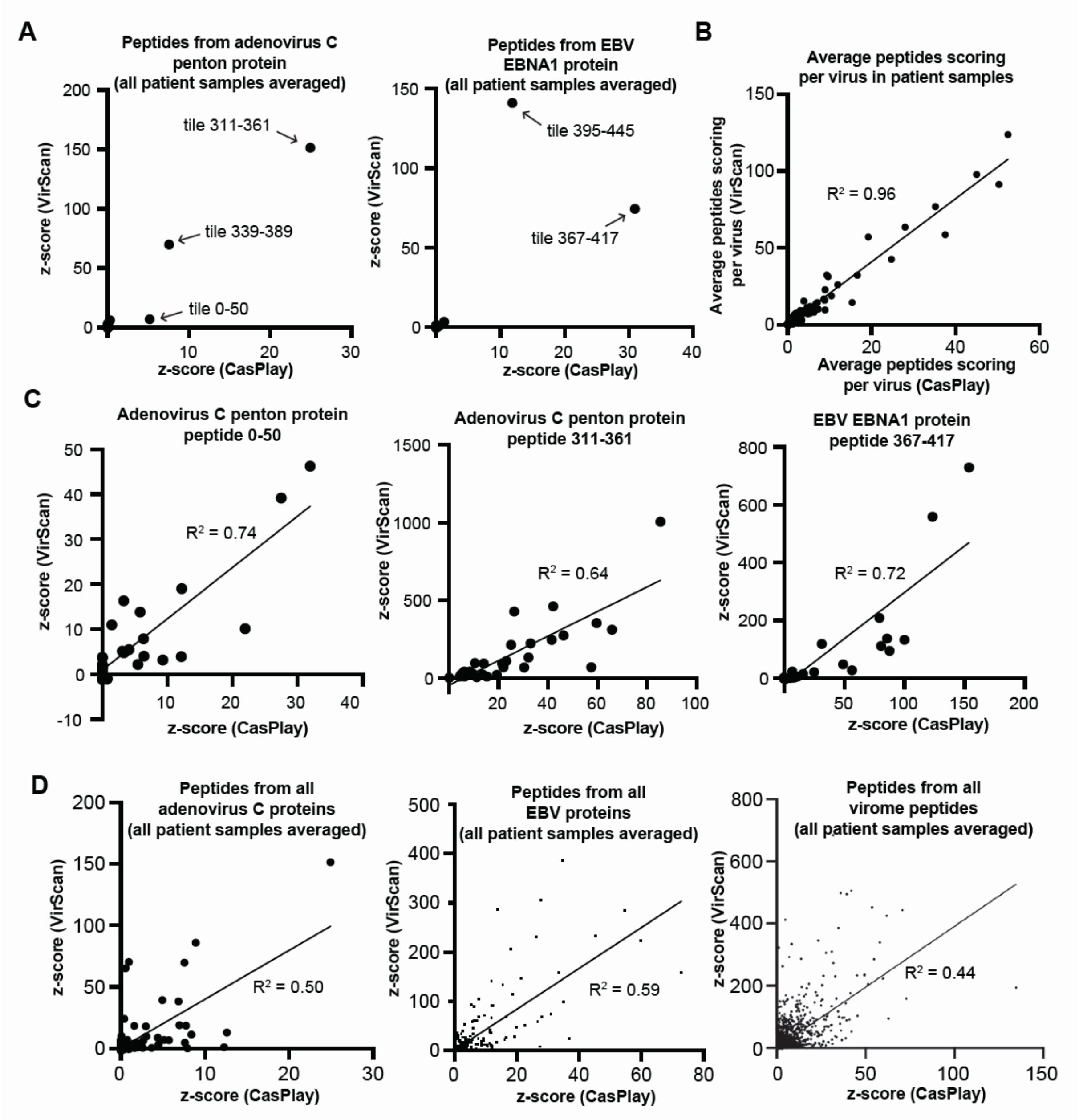

(**A**) Public epitopes from adenovirus C penton protein and EBV EBNA1 protein previously discovered by VirScan(Xu et al., 2015) were also uncovered by CasPlay (z-score ≥ 3.5). z-scores for each peptide from the annotated protein were averaged for 30 patient serum samples (with no suppositions about previous viral infection or vaccination status), measured in duplicate. (**B**) Average number of peptides scoring per

virus (for peptide z-score ≥ 3.5) across all 30 tested patient samples, compared between VirScan and CasPlay. (**C**) Correlation of peptide z-scores for representative public epitopes from 30 patient serum samples plotted individually, measured in duplicate. (**D**) Correlation of z-scores for each peptide derived from individual viruses or the entire virome averaged for 30 patient samples, compared between CasPlay and VirScan.

Supplementary Fig. 5: PICASSO-based application of dCas9-full length protein fusions, related to Fig. 5

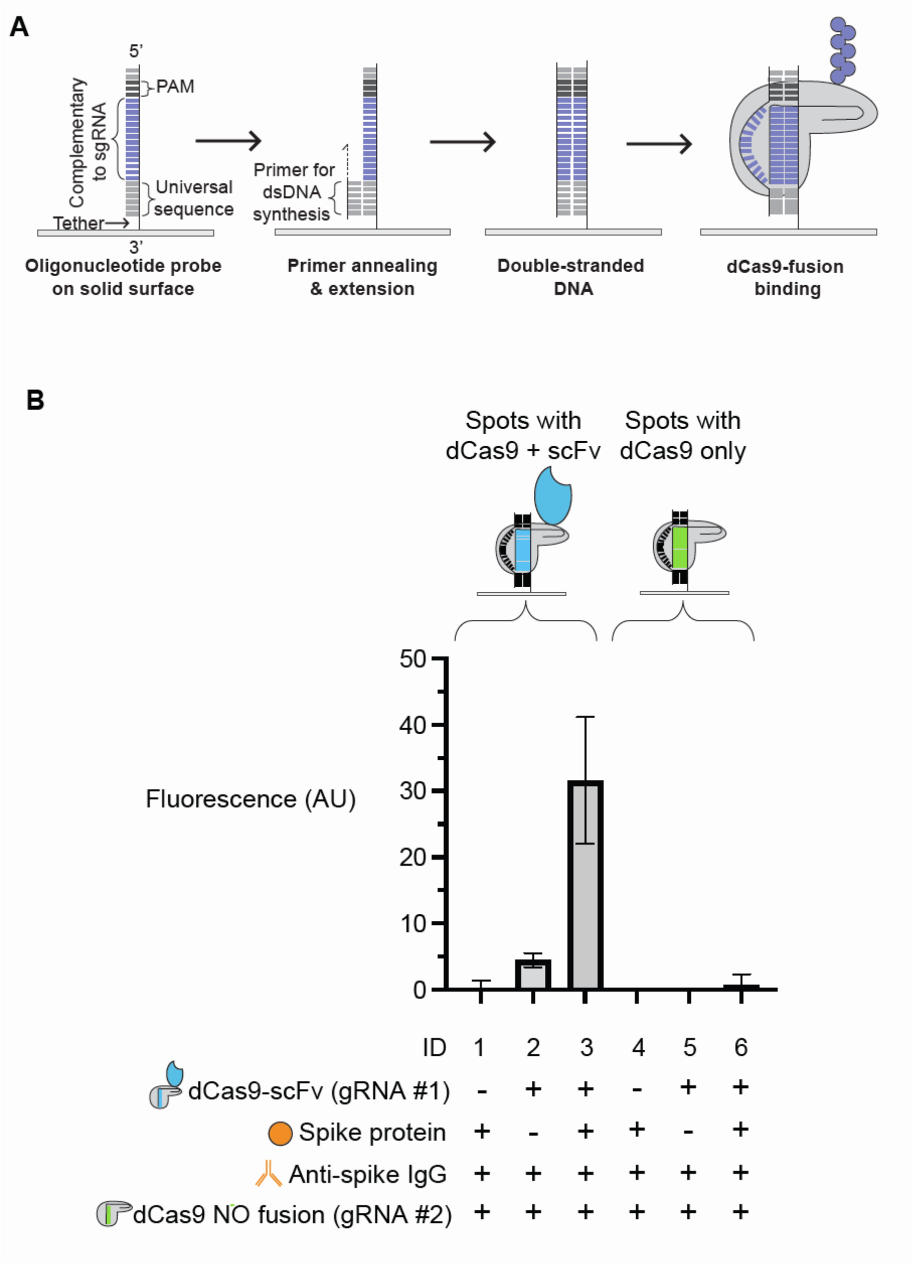

(**A**) Strategy for presenting a dCas9-fusion protein via PICASSO, adapted from Barber, et al., 2021(Barber et al., 2021). (**B**) dCas9-scFv fusion (anti-spike B1-182.1) presented via PICASSO enables fluorescence-based detection of corresponding analyte (spike protein). n = 6 replicate microarray features per condition.

Supplementary Table 1: Top 10 viruses with the most relative peptide hits by CasPlay and VirScan, related to Fig. 4

The average number of peptide hits (z-score ≥ 3.5) per virus per patient sample, reported as number of peptides or number of peptides as a percentage of the number of all peptides derived from that virus in the library by CasPlay and VirScan. Viruses are sorted by average peptide hits in CasPlay as a percentage of the viral proteome size, with viruses with fewer than 100 proteome peptides removed.

Virus	CasPlay - average peptide hits	VirScan - average peptide hits	Total peptides from virus encoded in library	CasPlay - peptide hits as percentage of encoded viral proteome	VirScan - peptide hits as percentage of encoded viral proteome
Human respiratory syncytial virus	52.5	123.7	781	6.7	15.8
Rhinovirus B	16.7	32.3	257	6.5	12.6
Rhinovirus A	35.2	76.9	660	5.3	11.7
Enterovirus B	45.1	97.8	1336	3.4	7.3
Enterovirus C	28.0	63.6	1009	2.8	6.3
Human parvovirus B19	4.3	8.9	169	2.6	5.3
Human herpesvirus 4	50.4	91.3	1980	2.5	4.6
Human adenovirus C	10.5	18.9	652	1.6	2.9
Human herpesvirus 1	24.8	42.7	1606	1.5	2.7
Influenza B virus	9.4	32.4	875	1.1	3.7
