# Supplementary material for: CasPlay provides a gRNA-barcoded CRISPR-based display platform for antibody repertoire profiling
Source: Cell Rep Methods. 2022 Oct 17;2(10):100318. doi: 10.1016/j.crmeth.2022.100318 (PMC9606310; doi:10.1016/j.crmeth.2022.100318)
Supplement: Document S2. Article plus supplemental information [file mmc5.pdf]

Cell Reports
Methods

Article

CasPlay provides a gRNA-barcoded CRISPR-based display platform for antibody repertoire profiling

Graphical abstract

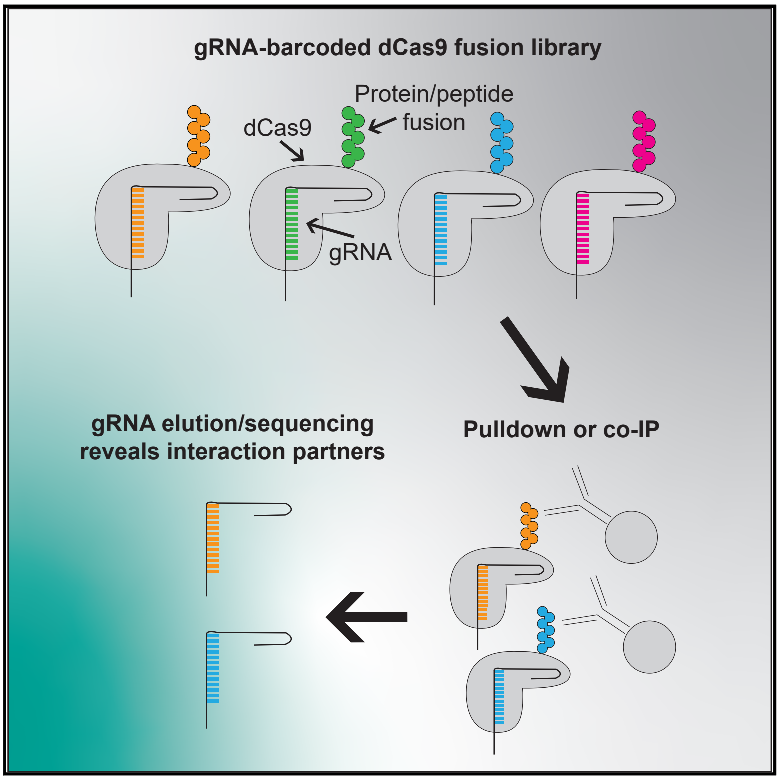

Highlights

- Peptide libraries fused to dCas9 are barcoded with unique gRNA sequences
- gRNA barcode sequencing enables protein binding characterization ("CasPlay")
- Antibody epitope mapping is performed for SARS-CoV-2 and across the human virome
- Full-length proteins are compatible with dCas9-based display

Authors

Karl W. Barber, Ellen Shrock, Stephen J. Elledge

Correspondence

selledge@genetics.med.harvard.edu

In brief

Barber et al. demonstrate that dCas9-fused peptide or protein libraries can be barcoded with gRNA sequences that can be analyzed by nucleic acid sequencing in a technique called "CasPlay." They carry out protein interaction studies by sequencing the gRNAs associated with interaction partners of a protein or proteins of interest.

Barber et al., 2022, Cell Reports Methods 2, 100318
October 24, 2022 © 2022 The Author(s).
https://doi.org/10.1016/j.crmeth.2022.100318

Cell Reports Methods

CellPress
OPEN ACCESS

Article

CasPlay provides a gRNA-barcoded CRISPR-based display platform for antibody repertoire profiling

Karl W. Barber,[1,2,3] Ellen Shrock,[1,2] and Stephen J. Elledge[1,2,4,*]
[1]Division of Genetics, Brigham and Women's Hospital, Howard Hughes Medical Institute, Boston, MA 02115, USA
[2]Department of Genetics, Harvard Medical School, Boston, MA 02115, USA
[3]Present address: Generate Biomedicines, 101 South St., Somerville, MA 02143, USA
[4]Lead contact
*Correspondence: selledge@genetics.med.harvard.edu
https://doi.org/10.1016/j.crmeth.2022.100318

MOTIVATION Protein display approaches such as phage and ribosome display have facilitated the performance of interaction studies using complex user-defined protein libraries catered toward an experimental question of interest. This study shows the utility and feasibility of generating complex dCas9-fused peptide libraries for performing protein interaction studies via high-throughput nucleic acid sequencing in a platform we name "CasPlay." In this work, we apply CasPlay to antibody epitope mapping experiments and demonstrate the display of synthetic antibodies by CasPlay for analyte detection. The methods and equipment required for these studies are widely available in standard molecular biology laboratories, further simplifying the execution of large-scale protein interaction discovery experiments.

SUMMARY

Protein display technologies link proteins to distinct nucleic acid sequences (barcodes), enabling multiplexed protein assays via DNA sequencing. Here, we develop Cas9 display (CasPlay) to interrogate customized peptide libraries fused to catalytically inactive Cas9 (dCas9) by sequencing the guide RNA (gRNA) barcodes associated with each peptide. We first confirm the ability of CasPlay to characterize antibody epitopes by recovering a known binding motif for a monoclonal anti-FLAG antibody. We then use a CasPlay library tiling the severe acute respiratory syndrome coronavirus 2 (SARS-CoV-2) proteome to evaluate vaccine-induced antibody reactivities. Using a peptide library representing the human virome, we demonstrate the ability of CasPlay to identify epitopes across many viruses from microliters of patient serum. Our results suggest that CasPlay is a viable strategy for customized protein interaction studies from highly complex libraries and could provide an alternative to phage display technologies.

INTRODUCTION

The application of CRISPR-Cas9 to gene editing has enabled routine and precise modification of DNA sequences in living cells (Cong et al., 2013; Jiang and Doudna, 2017; Mali et al., 2013). A catalytically inactive variant of Cas9 (dCas9) that retains the ability to specifically bind DNA sequences complementary to its associated guide RNA (gRNA) has been widely used to transcriptionally activate or silence gene targets (Bikard et al., 2013; Chavez et al., 2015; Gilbert et al., 2013, 2014; Qi et al., 2013). Additionally, the programmable nature of CRISPR systems to target user-defined genetic elements has further inspired *in vitro* applications for highly sensitive nucleic acid detection (English et al., 2019; Gootenberg et al., 2017).

In parallel with advances in technologies to modify and sequence DNA, high-throughput methods have also emerged to study complex protein populations. The use of *in vitro* protein display techniques has assisted in protein interaction discovery and binding characterization for use in basic research, clinical diagnostics, and therapeutic applications (Kretzschmar and Rüden, 2002; Wang and Yu, 2004). Phage and ribosome display platforms, which are common protein display strategies, operate by physically linking proteins to their encoding nucleic acid sequences, permitting protein selection and identification via DNA sequencing (Xu et al., 2015; Zhu et al., 2013). A common alternative reagent to expedite large-scale protein studies is the protein microarray, which contains thousands of proteins of interest spotted at discrete positions on a solid surface. However, traditional microarrays are expensive, difficult to fabricate, and non-resusable (Cretich et al., 2014; Hall et al., 2007; Hanash, 2003; Poetz et al., 2005).

To overcome some of these key limitations in display technologies, we recently combined aspects of nucleic acid barcoding and spatial arraying; in a technique termed peptide

This is an open access article under the CC BY license (http://creativecommons.org/licenses/by/4.0/).

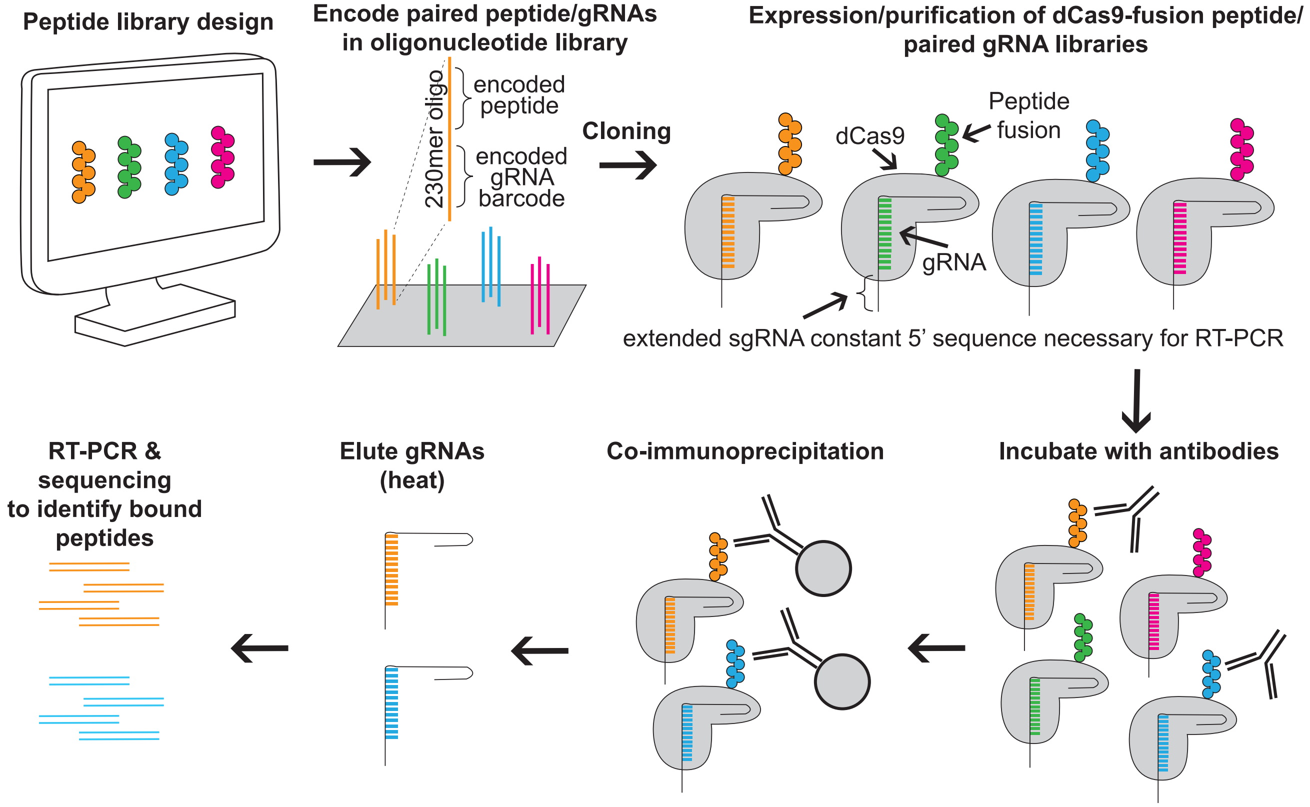

Figure 1. CasPlay library design and workflow

Customized peptide libraries are encoded in an oligonucleotide library, with each peptide sequence paired with a unique 20 bp nucleic acid sequence to be used as the gRNA barcode. The peptides are expressed in *E.* coli as fusions to dCas9 bound to unique gRNA barcode sequences and purified in a single batch. The gRNA sequences have a universal 5′ constant region to facilitate amplification by RT-PCR. This library is then used for immunoprecipitation experiments using human serum antibodies. Peptides bound to these antibodies are identified by nucleotide sequencing of the enriched gRNA barcodes.

immobilization via Cas9-mediated self-organization (PICASSO), customized peptide libraries fused to dCas9 are incubated with a double-stranded DNA (dsDNA) microarray, and each dCas9-fusion species localizes to the spot on the DNA microarray complementary to its gRNA barcode. In this manner, complex tailor-made peptide microarrays can be easily constructed via dCas9-fusion library self-assembly on a template DNA microarray for subsequent peptide library studies. However, we noted that PICASSO was not sensitive enough to detect certain antibody epitopes compared with phage display and required specialized microarray scanning capabilities unavailable in many laboratory settings.

Here, we demonstrate that dCas9-fusion libraries can be used for protein interaction studies using nucleic acid sequencing-based enrichment in a technique we term CasPlay. Using the same fundamental design principles as the gRNA-barcoded dCas9-fusion library assembly and purification, gRNA sequences are amplified by RT-PCR, and barcode abundances are tracked by next-generation sequencing (NGS). We perform immunoprecipitations using monoclonal antibodies and human serum samples, showing that CasPlay can be used to identify antibody specificities by detecting the enrichment of certain peptide species with gRNA barcode sequencing. We also perform an experiment to illustrate the compatibility of CasPlay with synthetic antibody presentation for analyte detection experiments. Overall, CasPlay is a versatile approach to catalog protein interactions with potential for diverse research and diagnostics applications.

RESULTS

CasPlay uncovers known anti-FLAG antibody peptide binding motif

To perform CasPlay experiments, we first design peptide sequences encoded on the same strand of DNA as an orthogonalized 20 nt barcode (Figure 1). The DNA library is then cloned in a single pool into an expression vector for the *E. coli*-based production of the peptide library as C-terminal fusions to dCas9 bound to a gRNA barcode that serves as a unique identifier for the peptide (Figure S1) (Barber et al., 2021). To facilitate sequencing of gRNAs for CasPlay, we added a 20 nt universal sequence to the 5′ end of the gRNAs to permit amplification by RT-PCR. We then use the CasPlay library for immunoprecipitation experiments by incubating the peptides with a collection of antibodies. gRNA sequences are then amplified and identified by NGS, and subsequent barcode enrichment analysis reveals peptides that were bound to the antibodies.

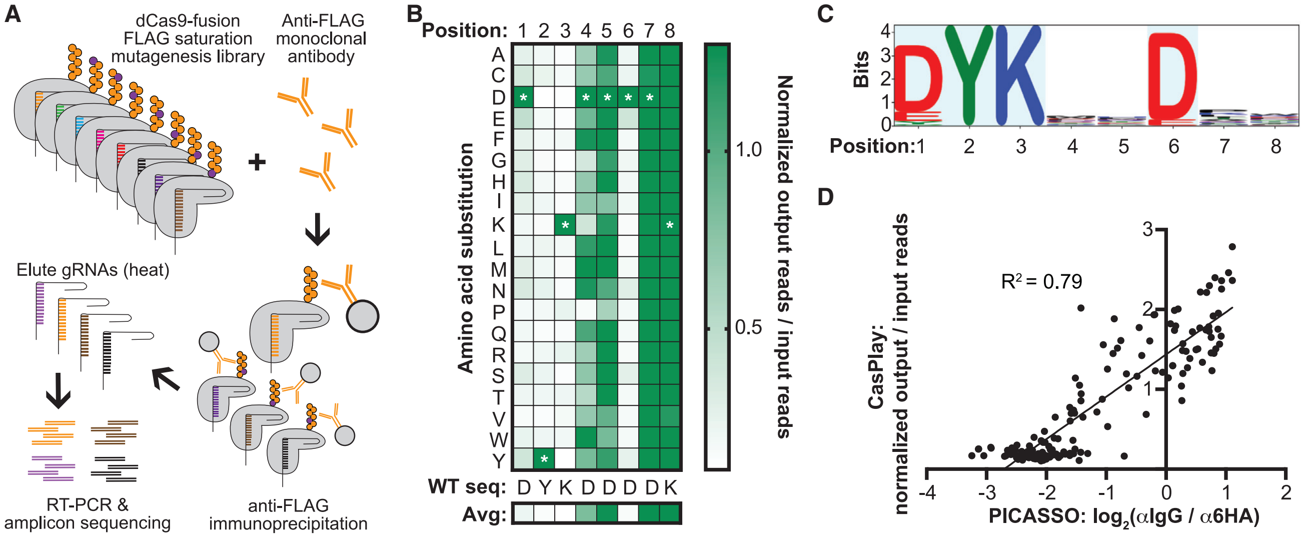

Figure 2. CasPlay experiments to map an antibody epitope with single amino acid resolution

(A) A FLAG peptide (DYKDDDDK) saturation mutagenesis library was produced for CasPlay in which every possible single amino acid substitution was performed along the length of the FLAG epitope, and each variant peptide was associated with a unique gRNA barcode.

(B) Sequencing-based enrichment analysis of gRNA barcodes revealed critical amino acid positions to coordinate M2 antibody binding to the FLAG peptide epitope. Enrichment data averaged across 4 gRNA barcode replicates and two independent experimental replicates.

(C) Enrichment of the DYKxxDxx motif for antibody binding was identified by sequence logo (Schneider and Stephens, 1990; Tareen and Kinney, 2020), consistent with previous studies (Layton et al., 2019).

(D) Sequence enrichment of FLAG variant peptides by CasPlay performed comparably with PICASSO, the microarray-based strategy for studying custom dCas9-fusion peptide libraries with fluorescence imaging readout (Barber et al., 2021).

As a first proof of concept of the CasPlay methodology to characterize antibody-epitope binding, we constructed a dCas9-displayed FLAG peptide saturation mutagenesis library encompassing all 152 possible single amino acid substitutions along the length of the FLAG epitope (DYKDDDDK; Figure 2A). We incubated this CasPlay library with the anti-FLAG M2 antibody, whose binding to the FLAG epitope has been extensively characterized (Layton et al., 2019). After immunoprecipitation, we amplified the gRNA sequences by RT-PCR and performed NGS to identify the barcodes of FLAG variant peptides that bound the M2 antibody (Figure 2B). We successfully recapitulated the known DYKxxDxx binding motif of the M2 antibody (Osada et al., 2009; Srila and Yamabhai, 2013), demonstrating the capability of CasPlay to identify amino acid residues that are critical to coordinate antibody binding (Figure 2C). The barcode enrichment analysis performed comparably to PICASSO experiments using the same dCas9-displayed FLAG variant library with M2 and a fluorescence-based microarray scanning assay (Barber et al., 2021) (R^2 = 0.79; Figure 2D), showing the ability of NGS-based gRNA sequencing to identify antibody-bound peptides.

Epitopes associated with SARS-CoV-2 infection or vaccination observed by CasPlay

We then constructed a CasPlay library consisting of 40-mer peptide tiles representing proteins from severe acute respiratory syndrome coronavirus 2 (SARS-CoV-2) (Figure 3A). We incubated this library with serum collected from patients previously infected with SARS-CoV-2 (n = 8) as well as human serum samples from before the beginning of the COVID-19 global pandemic (n = 8) and identified the peptides bound to patient antibodies using gRNA barcode sequencing. We were able to identify several regions from across the spike (S: 540–580; S: 764–804; S: 792–832; S: 1,128–1,168) and nucleocapsid (N: 148–188; N: 204–244; N: 380–420) proteins that were specifically enriched in at least two convalescent patient samples (barcode enrichment $\geq$3.5; Figure 3B). Comparing patient-matched results between CasPlay and PICASSO, CasPlay revealed one additional epitope that was not observed by PICASSO (N: 204–244), demonstrating enhanced sensitivity of the nucleic acid amplification-based approach (Figure S2). CasPlay identified all of the same shared epitopes observed using phage display experiments except one in the nucleocapsid protein (N: 92–132; Figure S2), potentially due to differences between the platforms in epitope context or displayed copy number.

Using the same CasPlay library, we also evaluated patient antibody reactivities elicited in response to SARS-CoV-2 mRNA vaccination (n = 8; Figure 3B). As anticipated, antibodies from vaccinated individuals exhibited binding to peptides from the spike protein but not the nucleocapsid, since the mRNA vaccine encodes only the spike protein (Turner et al., 2021). Furthermore, antibodies toward additional epitopes in the C-terminal domain of the S1 portion of the spike protein (S: 596–636; S: 652–692) were observed only in response to the vaccine and not in SARS-CoV-2-infected patients, consistent with previous studies (Garrett et al., 2022). CasPlay thereby enables nuanced characterization of differences in antibody repertoire between patients and could be used to stratify

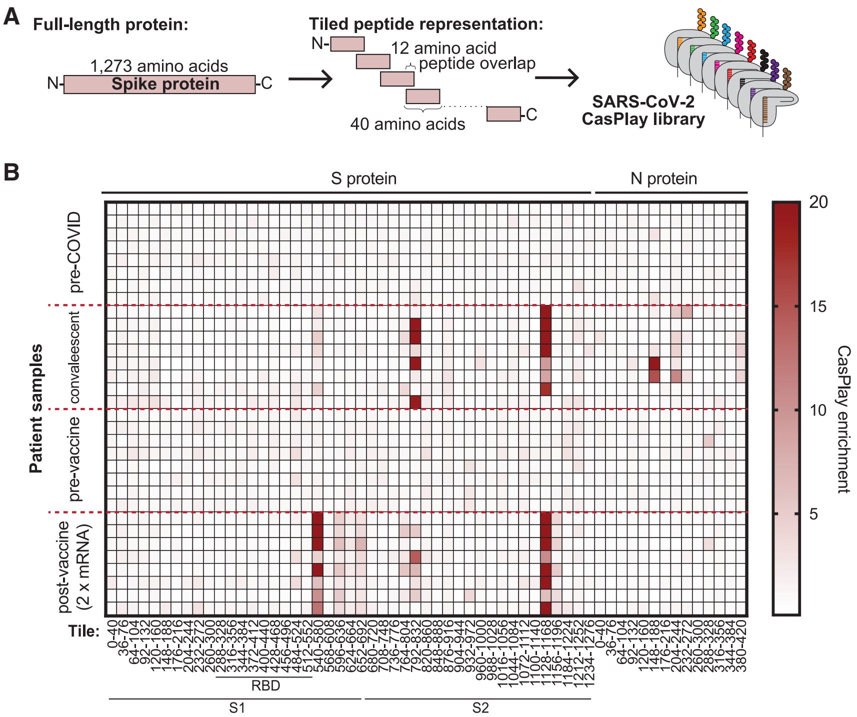

Figure 3. SARS-CoV-2 epitope mapping by CasPlay

(A) A peptide library tiling the SARS-CoV-2 proteome (excluding ORF1ab) from a previous study (Barber et al., 2021) was presented by CasPlay.

(B) CasPlay experiments were performed using serum samples from 8 samples collected prior to December 2020 ("pre-COVID"), 8 patients following COVID-19 infection ("convalescent"), 8 individuals prior to receiving a vaccine, and the same 8 individuals between 2 weeks and 3 months after receiving the second dose of an mRNA vaccine. gRNA barcode enrichment analysis by CasPlay revealed epitope regions in the spike and/or nucleocapsid proteins recognized by patient antibodies in convalescent and vaccinated patient samples. Enrichment is calculated as normalized output gRNA sequencing reads divided by pre-enriched normalized sequencing reads, with data averaged across 4 gRNA barcode replicates and two independent experimental replicates (see STAR methods for further details).

patients that were vaccinated against or naturally infected by SARS-CoV-2.

Human virome display by CasPlay enables antibody epitope localization simultaneously across many viruses

We then expanded the CasPlay library to encode a peptide-based representation of the known human virome, encompassing proteins from more than 243 viral species found in the UniProt database (Figure 4A). Based on our laboratory's previously described VirScan library (Shrock et al., 2020; Xu et al., 2015), we created a dCas9-displayed library of 50-mer peptides overlapping by 22 amino acids, tiled along the lengths of proteins derived from all viruses known to infect humans. Each of the 122,486 peptides was encoded in duplicate and paired with a unique gRNA barcode. Within the library, we also encoded peptides that are the targets of commercial monoclonal antibodies (FLAG, hemagglutinin [HA], and myc) for control experiments. NGS demonstrated that the CasPlay virome plasmid library was at least 96% complete, with approximately 96% of the gRNA barcodes falling within a 100-fold range of abundance (Figure S3A). Sequencing of the gRNAs after RT-PCR from the purified dCas9-fusion library exhibited more sequence skewing, with 73% of the barcodes falling within a 100-fold range of abundance and only evidence for at least 88% of the encoded sequences, though deeper sequencing analysis could reveal greater completion (Figure S3B). No correlation was observed between the gRNA barcode read counts in the plasmid library and the gRNAs from the purified protein library (Figure S3C), indicating that other peptide- or gRNA-specific factors affect final library abundance. Between independent preparations of the purified CasPlay library, relative barcode read counts were highly reproducible (R^2 = 0.97; Figure S3D).

To initially evaluate the performance of CasPlay for studies using the virome-wide library, we performed immunoprecipitations using anti-FLAG, anti-HA, and anti-myc monoclonal antibodies. gRNA barcode sequencing analysis revealed the selective enrichment of all 10 replicates of each of the anticipated epitopes for each tested antibody (Figure 4B). Within the entire CasPlay virome library (theoretical complexity of 245,002 barcodes), the barcodes for all replicate epitope tag peptides were ranked in the top 0.2% of Z scores in experiments using their corresponding target antibodies (Figure S3E). Since the HA epitope tag (YPYDVPDYA) is derived from a viral protein (influenza hemagglutinin), 98 viral peptides within the library (including replicates) also contained this sequence, of which 94 scored within the top 0.2% by Z score in the anti-HA immunoprecipitation. We observed that the lowest-ranking FLAG replicate peptide by Z score in the anti-FLAG immunoprecipitation experiment had a very low read count in the purified CasPlay "input" peptide library, suggesting that peptides that are of low abundance in the library may affect results interpretation for those sequences (Figure S3F).

We then performed immunoprecipitation experiments using the virome-wide CasPlay library with 30 human serum samples. As a benchmark of reproducibility, we looked at the total number of peptides that scored per virus in two patient-matched longitudinal samples and observed a strong correlation (R^2 = 0.97; Figure S3G) compared with two unrelated patient samples (R^2 = 0.42; Figure S3H). From our dataset, we looked for epitopes across common viruses that were widely recognized across patient samples (Figure 4C). We observed convergent antibody responses to peptides within the penton protein of adenovirus C (0: 50; 311–361; 339–389), Epstein-Barr virus (EBV) EBNA1 protein (367–417; 395–445), and respiratory syncytial virus (RSV)

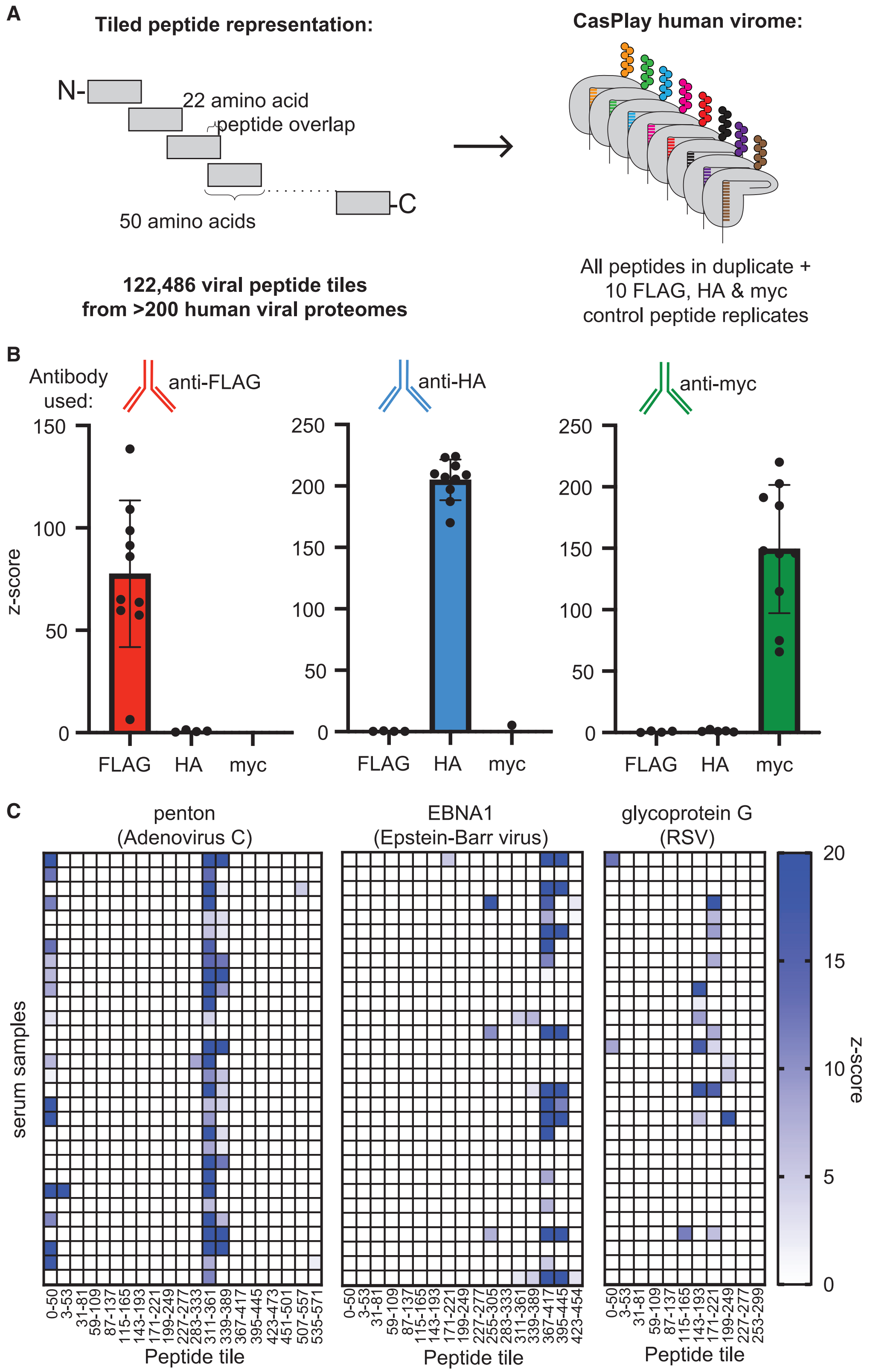

(legend on next page)

glycoprotein G (143–193; 171–221), all of which have been observed as public epitopes using VirScan (Figure S4A) (Xu et al., 2015).

To further evaluate CasPlay's performance, we performed comparative analysis using the same patient samples by VirScan. The average number of peptides scoring per virus in each patient sample (Z score $\geq$ 3.5) correlated very well between VirScan and CasPlay (R^2 = 0.96), though VirScan detected, on average, approximately 2-fold more peptide hits per virus (Figure S4B). The viruses with the most detected peptide-reactive antibodies per patient sample (including RSV, rhinoviruses, and enteroviruses) are ranked similarly between CasPlay and VirScan (Table S1). Across all tested patients, the Z scores for the public epitopes from adenovirus C and EBV correlated between CasPlay and VirScan (Figure S4C). Across the entire proteomes of adenovirus C and EBV as well as the virome library as a whole, the average patient Z score per peptide correlated more weakly between the two platforms (R^2 = 0.50, 0.59, and 0.44 respectively; Figure S4D). The decreased detection of certain peptide hits by CasPlay as well as differences in Z scores may be partially attributed to peptides with lower input counts in the library, as observed in Figure S3F.

Full-length functional synthetic antibodies are compatible with CasPlay

Finally, we sought to determine whether CasPlay is compatible with the display of longer folded proteins. To this end, we fused two classes of synthetic antibodies to dCas9: a nanobody recognizing β-catenin (Braun et al., 2016; Traenkle et al., 2015) and an scFv that binds the spike protein from SARS-CoV-2 (Wang et al., 2021) (Figure 5A). Each antibody was associated with a different gRNA barcode and purified from *E. coli*. To test the functionality of these antibodies, we immobilized target antigens (GST fused to the β-catenin epitope tag or recombinant SARS-CoV-2 spike protein) in separate wells of an adsorbent microplate. After applying a mixture of the two dCas9-fused antibodies, we washed away unbound protein and assessed the presence of each antibody using gRNA-specific primers for RT-PCR (Figure 5B). We observed retention of only the cognate synthetic antibody for each antigen using primers specific to the corresponding gRNA barcode (Figure 5C), showing that each antibody selectively bound its anticipated target and demonstrating the amenability of longer, folded proteins such as synthetic antibodies to CasPlay. We note that this type of antibody fusion can also be used for analyte detection via dCas9 immobilization by PICASSO (demonstrated in Figure S5).

DISCUSSION

In this study, we have illustrated the potential of dCas9-displayed peptides and proteins to be used for protein interaction studies using a simple gRNA-based sequencing readout. CasPlay is able to pinpoint amino acid positions within peptides coordinating antibody-epitope interactions as well as locate the epitopes of human serum antibodies within the context of larger proteins. We have also shown the potential of CasPlay using a very large (245,002) peptide library representing the human virome to identify epitopes across diverse viruses.

To improve results interpretation of this platform, future efforts will aim to design gRNA sequences computationally and/or empirically optimized for CasPlay. The decreased sensitivity of CasPlay compared with VirScan across many epitopes in the virome-wide library can potentially be attributed to low representation of certain dCas9-fusion peptides and gRNA barcodes in the "input" protein library, resulting in lack of barcode enrichment or skewed Z score calculation. These issues could possibly be further remediated by trying to better normalize the gRNA distribution in the library, deeper sequencing, or modulation of assay conditions and gRNA recovery and amplification. Additionally, some gRNAs may function poorly in the context of CasPlay due to suboptimal folding or dCas9 binding. Many groups have previously established criteria for effective gRNA design, primarily using genome editing efficacy as a readout (Chari et al., 2015; Doench et al., 2014; Moreno-Mateos et al., 2015). Future studies will take into account gRNA barcode performance in the experiments from this work as well as predicted sequence-specific gRNA secondary structures (Wong et al., 2015) in order to improve the design principles of gRNA libraries for CasPlay.

Still, differences in results between CasPlay and VirScan may persist due to differences in peptide copy number (one peptide is fused to dCas9 while multiple copies can be present on T7 phage [Ebrahimizadeh and Rajabibazl, 2014], which may increase antibody binding due to avidity) or spurious antibody-reactive epitopes due to engagement of residues flanking the peptide sequences. Future improvement of assay binding conditions may also further reduce the background of CasPlay to improve its sensitivity.

CasPlay provides a simple alternative approach to phage display, enabling gRNA sequencing-based enrichment analysis to track peptides that bind to proteins of interest. CasPlay has several advantageous features with respect to phage. While phage display platforms present proteins with variable copy numbers, CasPlay ensures that a single peptide or protein will be fused to each dCas9 molecule, eliminating potential confounding effects due to multivalency. This could be particularly important if one

Figure 4. CasPlay enables interrogation of antibodies on human virome scale

(A) 122,501-member tiled peptide-based representation of the human virome displayed by CasPlay based on peptide libraries from previous studies (Shrock et al., 2020; Xu et al., 2015). Each peptide was paired with an orthogonal gRNA barcode, with two separate barcodes assigned to each peptide (245,002 library members total). Epitope tags recognized by commercial monoclonal antibodies (e.g., FLAG, HA, myc) were also encoded as control peptides within the library.

(B) CasPlay control experiments using anti-FLAG, anti-HA, and anti-myc antibodies identified all ten corresponding control replicates and none of the off-target epitope tag peptides, within the context of the entire 245,002-member library. Error bars show SD centered at the mean.

(C) CasPlay was able to identify subregions of viral proteins commonly targeted by multiple patients (public epitopes) from within the 245,002-member library. Examples shown from adenovirus A, Epstein-Barr virus, and RSV. Z scores were averaged for gRNA barcode duplicates across two independent patient samples.

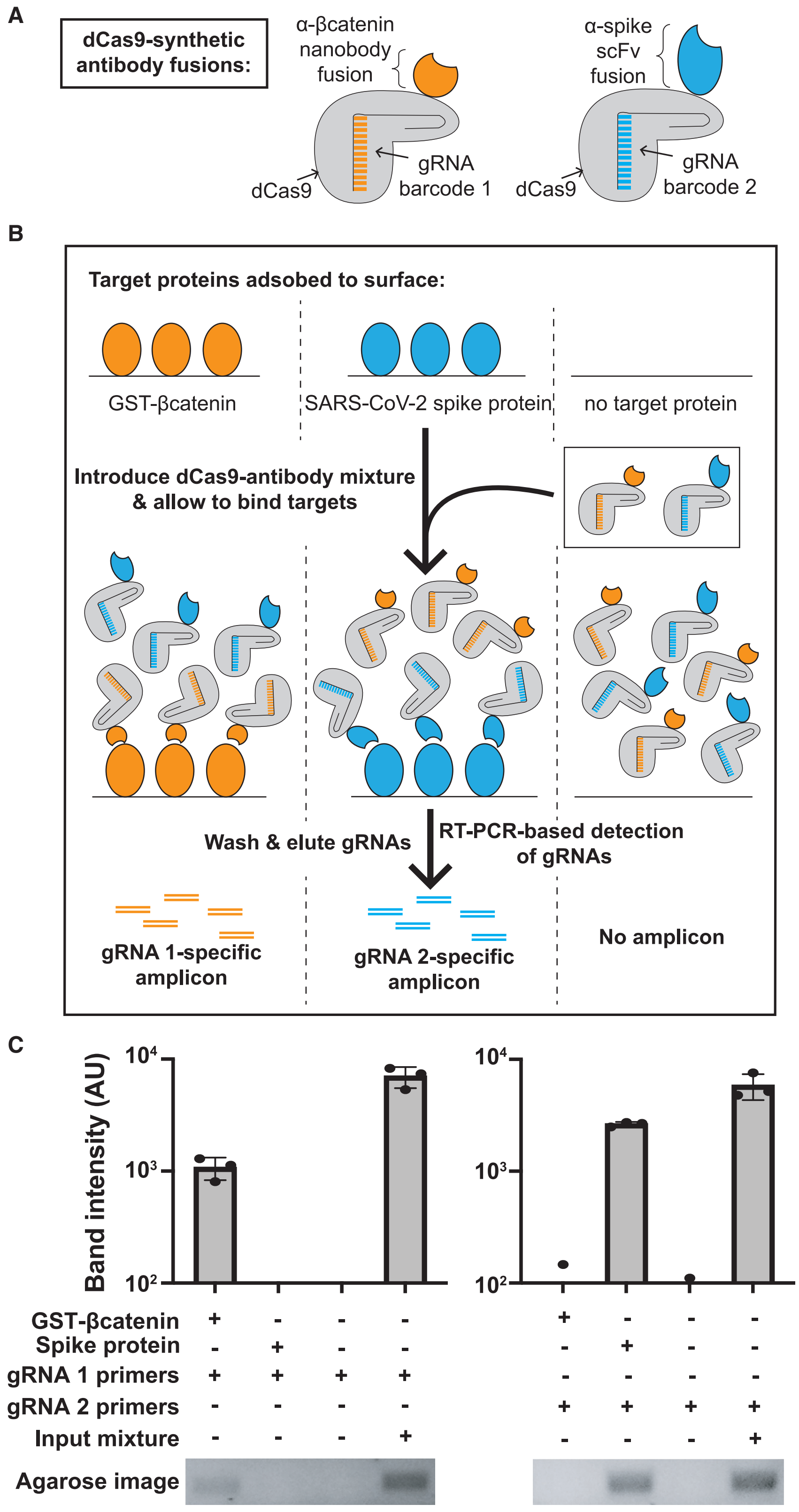

Figure 5. CasPlay is compatible with full-length protein display and applications

(A) Synthetic antibodies were expressed as C-terminal fusions to dCas9 with unique gRNA barcodes. A nanobody recognizing a peptide from β-catenin (Braun et al., 2016; Traenkle et al., 2015) and an scFv recognizing the spike protein from SARS-CoV-2 (Wang et al., 2021) were paired with unique gRNAs.

(B) Target antigens of the synthetic antibodies were adsorbed to a plate surface and then incubated with the dCas9-antibody fusions. Primers specific to the gRNA sequences were then used to RT-PCR the gRNAs associated with each antibody to detect binding of the antibody to its target antigen.

(C) Densitometry of RT-PCR amplicons on an agarose gel reveal specific retention and detection of each synthetic antibody only in the presence of its respective analyte, indicating synthetic antibody functionality. n = 3 independent replicates for each condition. Mean with SD plotted.

were assaying for the tightest binding antibodies and wishes to avoid avidity effects with a more linear readout. In addition, larger proteins often disrupt phage assembly, making them unsuitable for display; Cas9, as a monomeric protein, does not suffer from that disadvantage. CasPlay will be of particular interest to laboratories comfortable with recombinant protein expression and purification, obviating the need for phage library preparation techniques. Future efforts will aim to improve and rigorously characterize the sensitivity and specificity of CasPlay experiments and establish reliable scoring threshold criteria. Further collections of peptides associated with disease-related antibodies will also be curated and presented by CasPlay for potential diagnostic applications. Overall, CasPlay is a powerful technique that can be performed routinely by any molecular biology laboratory with protein purification capabilities for customized protein interaction studies.

Limitations of the study

Thus far, we have applied CasPlay primarily to the study of short peptide libraries, which lack much secondary and tertiary structure. However, we demonstrated in this work the successful dCas9-based display of two synthetic antibody fusions, suggesting that full-length protein libraries may be addressable by CasPlay. New approaches in library construction would need to be taken to assemble paired protein and gRNA sequences that cannot be readily synthesized on a short oligonucleotide. Continued improvements in DNA synthesis technologies will further facilitate preparation of CasPlay libraries, especially for longer proteins.

STAR★METHODS

Detailed methods are provided in the online version of this paper and include the following:

- KEY RESOURCES TABLE
- RESOURCE AVAILABILITY
  - Lead contact
  - Materials availability
  - Data and code availability
- EXPERIMENTAL MODEL AND SUBJECT DETAILS
  - Microbe strains
  - Human samples
- METHOD DETAILS
  - Design of CasPlay dCas9-peptide fusion libraries and synthetic antibody fusions
  - Oligonucleotide library design & cloning
  - CasPlay dCas9-fusion library expression and purification
  - CasPlay dCas9-fusion library precipitation and sequencing
  - CasPlay data analysis
  - dCas9-antibody fusion experiments
- QUANTIFICATION AND STATISTICAL ANALYSIS

SUPPLEMENTAL INFORMATION

Supplemental information can be found online at https://doi.org/10.1016/j.crmeth.2022.100318.

ACKNOWLEDGMENTS

K.W.B. received funding from Schmidt Science Fellows and the Jane Coffin Childs Memorial Fund for Medical Research. E.S. received funding from the National Science Foundation Graduate Research Fellowship Program. S.J.E. is an Investigator with the Howard Hughes Medical Institute.

AUTHOR CONTRIBUTIONS

K.W.B. and S.J.E. designed experiments and prepared this manuscript. E.S. provided SARS-CoV-2 vaccine cohort samples and aided in comparative analysis to VirScan data. K.W.B. performed all other experiments and analysis.

DECLARATION OF INTERESTS

K.W.B. and S.J.E. have filed a patent application with the US Patent and Trademark Office (US patent application no. 63/220,399) related to this work. S.J.E. is a founder of TScan Therapeutics, Maze Therapeutics, ImmuneID, and Mirimus. S.J.E. is a scientific advisory board member for Homology Medicines, TScan Therapeutics, ImmuneID, and Maze Therapeutics.

Received: July 2, 2022
Revised: September 6, 2022
Accepted: September 23, 2022
Published: October 17, 2022

REFERENCES

Barber, K.W., Shrock, E., and Elledge, S.J. (2021). CRISPR-based peptide library display and programmable microarray self-assembly for rapid quantitative protein binding assays. Mol. Cell *81*, 3650–3658.e5. https://doi.org/10.1016/j.molcel.2021.07.027.

Bikard, D., Jiang, W., Samai, P., Hochschild, A., Zhang, F., and Marraffini, L.A. (2013). Programmable repression and activation of bacterial gene expression using an engineered CRISPR-Cas system. Nucleic Acids Res. *41*, 7429–7437. https://doi.org/10.1093/nar/gkt520.

Braun, M.B., Traenkle, B., Koch, P.A., Emele, F., Weiss, F., Poetz, O., Stehle, T., and Rothbauer, U. (2016). Peptides in headlock – a novel high-affinity and versatile peptide-binding nanobody for proteomics and microscopy. Sci. Rep. *6*, 19211. https://doi.org/10.1038/srep19211.

Chari, R., Mali, P., Moosburner, M., and Church, G.M. (2015). Unraveling CRISPR-Cas9 genome engineering parameters via a library-on-library approach. Nat. Methods *12*, 823–826. https://doi.org/10.1038/nmeth.3473.

Chavez, A., Scheiman, J., Vora, S., Pruitt, B.W., Tuttle, M., P R Iyer, E., Lin, S., Kiani, S., Guzman, C.D., Wiegand, D.J., et al. (2015). Highly efficient Cas9-mediated transcriptional programming. Nat. Methods *12*, 326–328. https://doi.org/10.1038/nmeth.3312.

Cong, L., Ran, F.A., Cox, D., Lin, S., Barretto, R., Habib, N., Hsu, P.D., Wu, X., Jiang, W., Marraffini, L.A., and Zhang, F. (2013). Multiplex genome engineering using CRISPR/Cas systems. Science *339*, 819–823. https://doi.org/10.1126/science.1231143.

Cretich, M., Damin, F., and Chiari, M. (2014). Protein microarray technology: how far off is routine diagnostics? Analyst *139*, 528–542. https://doi.org/10.1039/c3an01619f.

Doench, J.G., Hartenian, E., Graham, D.B., Tothova, Z., Hegde, M., Smith, I., Sullender, M., Ebert, B.L., Xavier, R.J., and Root, D.E. (2014). Rational design of highly active sgRNAs for CRISPR-Cas9–mediated gene inactivation. Nat. Biotechnol. *32*, 1262–1267. https://doi.org/10.1038/nbt.3026.

Ebrahimizadeh, W., and Rajabibazl, M. (2014). Bacteriophage vehicles for phage display: biology, mechanism, and application. Curr. Microbiol. *69*, 109–120. https://doi.org/10.1007/s00284-014-0557-0.

English, M.A., Soenksen, L.R., Gayet, R.V., de Puig, H., Angenent-Mari, N.M., Mao, A.S., Nguyen, P.Q., and Collins, J.J. (2019). Programmable CRISPR-responsive smart materials. Science *365*, 780–785. https://doi.org/10.1126/science.aaw5122.

Garrett, M.E., Galloway, J.G., Wolf, C., Logue, J.K., Franko, N., Chu, H.Y., Matsen, F.A., and Overbaugh, J.M. (2022). Comprehensive characterization of the antibody responses to SARS-CoV-2 Spike protein finds additional vaccine-induced epitopes beyond those for mild infection. Elife *11*, e73490. https://doi.org/10.7554/elife.73490.

Gilbert, L.A., Larson, M.H., Morsut, L., Liu, Z., Brar, G.A., Torres, S.E., Stern-Ginossar, N., Brandman, O., Whitehead, E.H., Doudna, J.A., et al. (2013). CRISPR-mediated modular RNA-guided regulation of transcription in eukaryotes. Cell *154*, 442–451. https://doi.org/10.1016/j.cell.2013.06.044.

Gilbert, L.A., Horlbeck, M.A., Adamson, B., Villalta, J.E., Chen, Y., Whitehead, E.H., Guimaraes, C., Panning, B., Ploegh, H.L., Bassik, M.C., et al. (2014). Genome-scale CRISPR-mediated control of gene repression and activation. Cell *159*, 647–661. https://doi.org/10.1016/j.cell.2014.09.029.

Gootenberg, J.S., Abudayyeh, O.O., Lee, J.W., Essletzbichler, P., Dy, A.J., Joung, J., Verdine, V., Donghia, N., Daringer, N.M., Freije, C.A., et al. (2017). Nucleic acid detection with CRISPR-Cas13a/C2c2. Science *356*, 438–442. https://doi.org/10.1126/science.aam9321.

Hall, D.A., Ptacek, J., and Snyder, M. (2007). Protein microarray technology. Mech. Ageing Dev. *128*, 161–167. https://doi.org/10.1016/j.mad.2006.11.021.

Hanash, S. (2003). Disease proteomics. Nature *422*, 226–232. https://doi.org/10.1038/nature01514.

Hofacker, I.L. (2003). Vienna RNA secondary structure server. Nucleic Acids Res. *31*, 3429–3431. https://doi.org/10.1093/nar/gkg599.

Jiang, F., and Doudna, J.A. (2017). CRISPR–Cas9 structures and mechanisms. Annu. Rev. Biophys. *46*, 505–529. https://doi.org/10.1146/annurev-biophys-062215-010822.

Kretzschmar, T., and von Rüden, T. (2002). Antibody discovery: phage display. Curr. Opin. Biotechnol. *13*, 598–602. https://doi.org/10.1016/s0958-1669(02)00380-4.

Larman, H.B., Zhao, Z., Laserson, U., Li, M.Z., Ciccia, A., Gakidis, M.A.M., Church, G.M., Kesari, S., LeProust, E.M., Solimini, N.L., and Elledge, S.J. (2011). Autoantigen discovery with a synthetic human peptidome. Nat. Biotechnol. *29*, 535–541. https://doi.org/10.1038/nbt.1856.

Layton, C.J., McMahon, P.L., and Greenleaf, W.J. (2019). Large-scale, quantitative protein assays on a high-throughput DNA sequencing chip. Mol. Cell *73*, 1075–1082.e4. https://doi.org/10.1016/j.molcel.2019.02.019.

Mali, P., Yang, L., Esvelt, K.M., Aach, J., Guell, M., DiCarlo, J.E., Norville, J.E., and Church, G.M. (2013). RNA-guided human genome engineering via Cas9. Science *339*, 823–826. https://doi.org/10.1126/science.1232033.

Martin, M. (2011). Cutadapt removes adapter sequences from high-throughput sequencing reads. EMBnet. J. *17*, 10–12. https://doi.org/10.14806/ej.17.1.200.

Mina, M.J., Kula, T., Leng, Y., Li, M., de Vries, R.D., Knip, M., Siljander, H., Rewers, M., Choy, D.F., Wilson, M.S., et al. (2019). Measles virus infection diminishes preexisting antibodies that offer protection from other pathogens. Science *366*, 599–606. https://doi.org/10.1126/science.aay6485.

Moreno-Mateos, M.A., Vejnar, C.E., Beaudoin, J.-D., Fernandez, J.P., Mis, E.K., Khokha, M.K., and Giraldez, A.J. (2015). CRISPRscan: designing highly efficient sgRNAs for CRISPR/Cas9 targeting in vivo. Nat. Methods *12*, 982–988. https://doi.org/10.1038/nmeth.3543.

Osada, E., Shimizu, Y., Akbar, B.K., Kanamori, T., and Ueda, T. (2009). Epitope mapping using ribosome display in a reconstituted cell-free protein synthesis system. J. Biochem. *145*, 693–700. https://doi.org/10.1093/jb/mvp027.

Poetz, O., Ostendorp, R., Brocks, B., Schwenk, J.M., Stoll, D., Joos, T.O., and Templin, M.F. (2005). Protein microarrays for antibody profiling: specificity and affinity determination on a chip. Proteomics *5*, 2402–2411. https://doi.org/10.1002/pmic.200401299.

Qi, L.S., Larson, M.H., Gilbert, L.A., Doudna, J.A., Weissman, J.S., Arkin, A.P., and Lim, W.A. (2013). Repurposing CRISPR as an RNA-guided platform for sequence-specific control of gene expression. Cell *152*, 1173–1183. https://doi.org/10.1016/j.cell.2013.02.022.

Schneider, T.D., and Stephens, R.M. (1990). Sequence logos: a new way to display consensus sequences. Nucleic Acids Res. *18*, 6097–6100. https://doi.org/10.1093/nar/18.20.6097.

Shrock, E., Fujimura, E., Kula, T., Timms, R.T., Lee, I.-H., Leng, Y., Robinson, M.L., Sie, B.M., Li, M.Z., Chen, Y., et al. (2020). Viral epitope profiling of COVID-19 patients reveals cross-reactivity and correlates of severity. Science *370*, eabd4250. https://doi.org/10.1126/science.abd4250.

Srila, W., and Yamabhai, M. (2013). Identification of amino acid residues responsible for the binding to Anti-FLAG^{TM} M2 antibody using a phage display combinatorial peptide library. Appl. Biochem. Biotechnol. *171*, 583–589. https://doi.org/10.1007/s12010-013-0326-8.

Tareen, A., and Kinney, J.B. (2020). Logomaker: beautiful sequence logos in Python. Bioinformatics *36*, 2272–2274. https://doi.org/10.1093/bioinformatics/btz921.

Traenkle, B., Emele, F., Anton, R., Poetz, O., Haeussler, R.S., Maier, J., Kaiser, P.D., Scholz, A.M., Nueske, S., Buchfellner, A., et al. (2015). Monitoring interactions and dynamics of endogenous beta-catenin with intracellular nanobodies in living cells*[S]. Mol. Cell. Proteomics *14*, 707–723. https://doi.org/10.1074/mcp.m114.044016.

Turner, J.S., O'Halloran, J.A., Kalaidina, E., Kim, W., Schmitz, A.J., Zhou, J.Q., Lei, T., Thapa, M., Chen, R.E., Case, J.B., et al. (2021). SARS-CoV-2 mRNA vaccines induce persistent human germinal centre responses. Nature *596*, 109–113. https://doi.org/10.1038/s41586-021-03738-2.

Wang, L.-F., and Yu, M. (2004). Epitope identification and discovery using phage display libraries: applications in vaccine development and diagnostics. Curr. Drug Targets *5*, 1–15. https://doi.org/10.2174/1389450043490668.

Wang, L., Zhou, T., Zhang, Y., Yang, E.S., Schramm, C.A., Shi, W., Pegu, A., Oloniniyi, O.K., Henry, A.R., Darko, S., et al. (2021). Ultrapotent antibodies against diverse and highly transmissible SARS-CoV-2 variants. Science *373*, eabh1766. https://doi.org/10.1126/science.abh1766.

Wong, N., Liu, W., and Wang, X. (2015). WU-CRISPR: characteristics of functional guide RNAs for the CRISPR/Cas9 system. Genome Biol. *16*, 218. https://doi.org/10.1186/s13059-015-0784-0.

Xu, G.J., Kula, T., Xu, Q., Li, M.Z., Vernon, S.D., Ndung'u, T., Ruxrungtham, K., Sanchez, J., Brander, C., Chung, R.T., et al. (2015). Comprehensive serological profiling of human populations using a synthetic human virome. Science *348*, aaa0698. https://doi.org/10.1126/science.aaa0698.

Zhu, J., Larman, H.B., Gao, G., Somwar, R., Zhang, Z., Laserson, U., Ciccia, A., Pavlova, N., Church, G., Zhang, W., et al. (2013). Protein interaction discovery using parallel analysis of translated ORFs (PLATO). Nat. Biotechnol. *31*, 331–334. https://doi.org/10.1038/nbt.2539.

Zorita, E., Cuscó, P., and Filion, G.J. (2015). Starcode: sequence clustering based on all-pairs search. Bioinformatics *31*, 1913–1919. https://doi.org/10.1093/bioinformatics/btv053.

STAR★METHODS

KEY RESOURCES TABLE

REAGENT or RESOURCE	SOURCE	IDENTIFIER
Antibodies		
Anti-6His, rabbit	Cell Signaling	D3I1O; RRID: AB_2744546
Anti-HA, rabbit	Cell Signaling	C29F4; RRID: AB_1549585
Anti-myc, rabbit	Abcam	ab9106; RRID: AB_307014
Anti-FLAG M2, mouse	Millipore Sigma	F1804; RRID: AB_262044
SARS-CoV-2 spike protein	Sino Biological	40589-V08H4
anti-SARS-CoV-2 spike CR3022 human IgG antibody	Cell Signaling	37475; RRID: AB_2848080
Alexa 647-conjugated anti-human IgG Fc antibody	Biolegend	410714; RRID: AB_2728444
Bacterial and virus strains		
T7 Express lysY Competent *E. coli*	NEB	C3010I
Electromax DH10B *E. coli*	ThermoFisher	18290015
Oligonucleotides		
SurePrint Oligonucleotide Libraries listed in Supplemental File 2	This paper (synthesized by Agilent)	G7220A
Oligonucleotide microarrays as PICASSO templates	This paper (synthesized by CustomArray/Genscript)	N/A
Biological samples		
Human serum samples	Shrock et al. (2020) and this paper	N/A
Recombinant DNA		
pgRNA-bacteria	Qi et al. (2013)	Addgene #44251
pdCas9-bacteria plasmid	Qi et al. (2013)	Addgene #44249
dCas9-fusion + sgRNA library expression vector (see Supplementary File 2)	Barber et al. (2021)	Addgene #171798
dCas9-fusion + sgRNA expression scaffold (see Supplementary File 2)	Barber et al. (2021)	Addgene #171799
dCas9-nanobody BC2-Nb	This paper	Addgene #186420
dCas9-ultrapotent B1-182.1	This paper	Addgene #186421
GST-beta catenin tag	This paper	Addgene #186422
Software and algorithms		
GenePix Pro 7	Molecular Devices	N/A
Other		
GenePix 4300A microarray scanner	Molecular Devices	GENEPIX 4300

RESOURCE AVAILABILITY

Lead contact

Further information and requests for resources and reagents should be directed to and will be fulfilled by the lead contact, Stephen J. Elledge (selledge@genetics.med.harvard.edu).

Materials availability

All experimental materials are available from the authors upon reasonable request. Plasmids generated for this study have been deposited to Addgene (dCas9-nanobody BC2-Nb: Addgene #186420; dCas9-ultrapotent B1-182.1: Addgene #186421; GST-beta catenin tag: Addgene #186422).

Data and code availability

- Read counts of gRNA barcodes identified by NGS for all experiments are provided as supplemental tables.
- This paper does not report original code.
- Any additional information required to reanalyze the data reported in this paper is available from the lead contact upon request.

EXPERIMENTAL MODEL AND SUBJECT DETAILS

Microbe strains

Plasmid and plasmid library cloning was performed in ElectroMAX DH10B *E. coli* cells (Thermo Fisher) grown at 37°C dCas9-fusion libraries were expressed in T7 Express lysY Competent *E. coli* (High Efficiency, NEB) grown at 37°C. Further information about expression conditions are included in the "method details" section.

Human samples

COVID-19 convalescent samples and healthy controls were collected and analyzed by VirScan in previous studies (Shrock et al., 2020). Eight blood samples (deemed to be exempt research in Mass General Brigham IRB approved protocol 2020P001619) were used for the pre- and post-vaccine cohort analyzed in this study. No identifying information besides vaccine status was collected.

METHOD DETAILS

Design of CasPlay dCas9-peptide fusion libraries and synthetic antibody fusions

The dCas9-fusion peptides used for anti-FLAG M2 antibody epitope binding characterization and for targeted SARS-CoV-2 epitope mapping experiments were designed and described previously (Barber et al., 2021).

The human virome peptide library was designed based on previous phage display libraries (120,396 peptides from viruses that infect humans (Xu et al., 2015) plus 1,794 coronavirus-derived peptides (Shrock et al., 2020)). In these prior studies, 56mer peptides tiling viral proteins with 28 amino acid overlap between adjacent tiles were presented on T7 phage. These peptides were used as the basis for the design of the 50mer peptides used in CasPlay, centered around the same residues as the 56mer peptides (i.e. the peptides presented by CasPlay were 3 amino acids shorter on both the N- and C-termini, and adjacent tiles overlapped by 22 amino acids). Additional 50mer peptides were included to encompass the N- and C-termini of each protein. 292 peptides representing SARS-CoV-2 variants with United States Centers for Disease Control and Prevention designations "being monitored" and "of concern" (https://www.cdc.gov/coronavirus/2019-ncov/variants/variant-classifications.html) as of January 2022 were also included in the library; for these peptides, the amino acid substitutions and deletions occurring in the viral variant proteins were incorporated in the corresponding peptide tiles, such that the register of the tiles was not altered from the original SARS-CoV-2 library peptide tiles to enable binding comparisons between variant peptides. Control peptide epitope tags, including HA, myc and FLAG, were also included in the library.

Oligonucleotide library design & cloning

The CasPlay-compatible 50mer viral peptides were codon optimized for expression in *E. coli* by mimicking natural codon frequency with rare codons removed (Xu et al., 2015). Each peptide was encoded in duplicate (separately codon optimized), with the exception of the monoclonal epitope tag controls (HA, myc and FLAG), which were encoded with 10 replicates. Each peptide was associated with a unique, synthetic gRNA sequence that differed from every other gRNA sequence by at least 1 base pair within the first 10 bases from the 3′ end of the spacer sequence; the remaining 10 bases were randomized, with the stipulations that extraneous protospacer adjacent motifs ("CCN") and polyT sequences ("TTTT") be removed. Each gRNA sequence was additionally ensured to have a minimum Levenshtein distance of 3 from every other sequence within the library (Zorita et al., 2015). gRNA spacer sequences with the lowest degree of predicted secondary structure (Hofacker, 2003) were then selected from this set. Each peptide replicate was associated with a unique gRNA sequence.

The oligonucleotides contained the following, from 5′ to 3': homology arm for Gibson assembly (5′-GAGGAGGTTCTCGATCG-3′); peptide-encoding region; SalI restriction site; randomized bases to make total oligo length 230 bp (only included for peptides shorter than 50 amino acids, such as epitope tag controls); additional A base; XhoI restriction site; additional A base; SpeI restriction site; gRNA spacer sequence; homology arm for Gibson assembly (5′-GTTTTAGAGCTAGAAATAGCAAG-3′). The 245,004 230mer oligonucleotides were synthesized across two equal-sized pools by Agilent Technologies.

Primers complementary to the homology arms within the oligonucleotides were used for library amplification using Q5 polymerase (NEB) on a 50 μL scale with 100 fmol of the oligonucleotide library template, 59°C annealing temperature, and 60 s extension time with a total of 10 amplification cycles. PCR products were desalted using a PCR clean-up spin column (Machery-Nagel). The library amplicon was introduced into the BsaI/PvuI-digested precursor vector (Addgene #171798) using 80 ng vector backbone and 20 ng amplified oligonucleotide library insert, in a 10 μL total HiFi reaction (NEB). After incubation at 50°C for 1 h, the DNA was desalted using 0.7x Ampure XP beads (Beckman Coulter) and transformed into 20 μL ElectroMAX DH10B cells (Thermo Fisher). This was

performed in quadruplicate for each of the two Agilent oligonucleotide pools. After 1 h recovery in 1 mL SOC at 37°C, cells were spread on 15 cm LB + 100 μg/mL carbenicillin plates. The following morning, cells were scraped and miniprepped to harvest the vector libraries.

For the second library subcloning step (for tiled human virome and targeted FLAG saturation mutagenesis and SARS-CoV-2 epitope libraries), an insert encoding a 6His tag, stop codon, transcriptional terminator, chloramphenicol resistance marker, T7 promoter for gRNA expression, and 5′ gRNA constant region for RT-PCR-based amplification was amplified from a previously described vector (Addgene #171799) (Barber et al., 2021) using the following primers: 5′-GGAAGAGTCGACCACCATC-3′ and 5′-CAACCAACACTAGTACGTAGTCTGTACCTGATCTCTATAGTGAGTCGTATTAGATCTTTAGGACGTCGATATCTG-3′. This insert amplicon and the precursor plasmid library detailed above were digested with SalI and SpeI. 100 ng of the digested library backbone was ligated with 50 ng of digested insert using T4 DNA ligase (NEB). 10 replicate ligation reactions were performed for each library. The ligations were desalted using 0.7x Ampure beads (Beckman Coulter) and transformed into 20 μL ElectroMAX DH10B cells (Thermo Fisher). After 1 h recovery in 1 mL SOC at 37°C, cells were spread on 15 cm LB + 100 μg/mL carbenicillin +50 μg/mL chloramphenicol plates. The following morning, cells were scraped and miniprepped to harvest the vector libraries.

CasPlay dCas9-fusion library expression and purification

100 ng of the final plasmid library was transformed into T7 Express *lysY E. coli* (NEB). After 1 h recovery in 1 mL LB at 37°C, cells were inoculated into 50 mL LB + 100 μg/mL carbenicillin +50 μg/mL chloramphenicol and grown at 37°C for 16 h. Cells were then diluted to OD_{600} = 0.2 in 250 mL LB + 100 μg/mL carbenicillin +50 μg/mL chloramphenicol, shaking at 225 rpm. The four sublibraries for the targeted FLAG saturation mutagenesis experiments were combined at this stage. Separately, the four sublibraries for the SARS-CoV-2 epitope mapping experiments were also combined at this stage. The two-halves of the human virome library were not combined and were isolated in parallel. When cells reached OD_{600} = 0.8, dCas9-fusion peptide and gRNA expression were induced with 100 ng/mL anhydrotetracycline (ATC) and 0.1 mM IPTG, respectively. Cells were grown at 37°C, 225 rpm for an additional 4 h. Cells were harvested by centrifugation and pellets were stored at −80°C for at least 12 h.

Once thawed, cell pellets were resuspended in 12.5 mL lysis buffer containing 50 mM Tris pH 7.5, 500 mM NaCl, 10% glycerol, 100 μM DTT, 5 μL rLysozyme solution (Millipore Sigma), 25 μL benzonase (90% purity, Millipore Sigma), 1x BugBuster (Millipore Sigma), and 1x protease inhibitors (cOmplete EDTA-free, Millipore Sigma), rotating at 25°C for 30 min. Clarified lysates were incubated with 250 μL bed volume equilibrated Ni-NTA agarose (Qiagen) for 30 min at 23°C, rotating end-over-end. Resin was washed twice with 2.5 mL wash buffer (50 mM Tris pH 7.4, 500 mM NaCl, 10% glycerol, 100 μM DTT, 20 mM imidazole), and dCas9-fusions complexed with gRNAs were eluted using 2 × 250 μL elution buffer (50 mM Tris pH 7.4, 500 mM NaCl, 10% glycerol, 100 μM DTT, 500 mM imidazole). Eluates were passed through a 45 μm filter and buffer exchanged using a 100 kDa molecular weight cutoff Amicon Ultra-4 centrifugal filter (Millipore Sigma) with storage buffer (50 mM Tris pH 7.4, 150 mM NaCl, 10% glycerol, 1 mM DTT). Protein concentration was estimated by A_{260} and protein was stored at −20°C.

CasPlay dCas9-fusion library precipitation and sequencing

Convalescent serum and samples from before December 2020 were described previously (Shrock et al., 2020). Deidentified longitudinal vaccine cohort samples of individuals with no known prior SARS-CoV-2 infection were collected prior to SARS-CoV-2 vaccination as well as between two weeks and three months after administration of the second dose of either Pfizer or Moderna mRNA vaccine. Patient serum samples were diluted 1:50 in PBS. 10 μL diluted serum was transferred into a 96 well plate and mixed with 20 ng of dCas9-6His bound to a gRNA lacking the 5′ overhang necessary for RT-PCR, 1% BSA (BSA, w/v), and 250 μg/mL salmon sperm DNA (ThermoFisher), diluted to 60 μL total volume in TBST. For experiments using monoclonal antibodies, 1 μg of antibody was used (anti-FLAG M2 Millipore Sigma F1804; anti-HA Cell Signaling C29F4; anti-myc Abcam ab9106). Samples were incubated mixing end-over-end for 30 min at ambient temperature. Approximately 1 μg dCas9-fusion library was then mixed with each sample. For experiments using the tiled human virome, the two purified dCas9-fusion library subpools were combined prior to addition to the serum samples. Control experiments lacking serum or monoclonal antibodies were also performed to assess dCas9-fusion library background binding to the beads. Samples were then incubated at ambient temperature mixing end-over-end for 1 h. 20 μL of protein A Dynabeads (ThermoFisher) and 20 μL of protein G Dynabeads (ThermoFisher) were then added to each sample. Samples were then incubated for 16 h at 4°C, rotating end-over-end. Samples were then washed with 6 × 100 μL TBST on a magnet plate. Beads were then resuspended in 10 μL water and heated to 95°C for 5 min to elute gRNAs. 6.5 μL of eluate was used for reverse transcription with Super-Script IV (ThermoFisher) using the manufacturer-suggested protocol on a 0.5x scale with primer 5′-GCACCGACTCGGTGCCACTTTTTC-3′. Samples were then amplified using primers 5′-AGATCAGGTACAGACTACGTACTAG-3′ and 5′-GCACCGACTCGGTGC-3′ with Q5 polymerase (NEB) at 65°C with 20 s extension time and 45 amplification cycles. Adapters for pooled Illumina sequencing were appended by PCR as previously described (Larman et al., 2011; Xu et al., 2015). Pooled gRNA amplicons were sequenced using an Illumina NextSeq 500 with approximately 2 million single-end 150 bp reads per sample.

CasPlay data analysis

From NGS reads of gRNA amplicons, constant regions on sequencing reads surrounding the gRNA barcodes were removed using Cutadapt v2.5 (Martin, 2011). Raw read counts were obtained by assigning each sequencing read to an encoded gRNA barcode if the sequence was a perfect match to an anticipated barcode (20/20 correct bases) and associating the sequence with its paired peptide.

For analysis of CasPlay FLAG saturation mutagenesis and SARS-CoV-2 peptide libraries, gRNA barcode normalized counts after immunoprecipitation were divided by the normalized read counts in the purified "input" dCas9-fusion library to calculate enrichment. Enrichment values for each replicate peptide were averaged for all gRNA barcodes with at least 50 or 100 raw read counts in the "input" sample in the FLAG and SARS-CoV-2 experiments, respectively. For SARS-CoV-2 experiments in Figure 3, results were further normalized by dividing the enrichment score of each peptide by the average score of the same peptide from the pre-COVID samples. Values for all analysis were averaged for two independent antibody sample replicates. For the CasPlay virome-wide library, gRNA barcodes from the purified "input" protein libraries used for experiments were rank-ordered based on read counts and divided into 300-member bins with the top and bottom 5% of sequences excluded. gRNA barcodes with zero readcounts in the input library were assigned a pseudocount of 0.999. A *Z* score for each peptide in experimental samples was then calculated based on the peptide sequence read count and the mean and SD of read counts within its assigned bin (Mina et al., 2019). To increase stringency of analysis, *Z* score averages were obtained only for peptides with *Z* score $\geq$ 2 for either two peptide barcode replicates or two patient sample replicates; all other average z-scores were set to zero. Sequence alignments, *Z* score calculations, and data analysis were performed using *ad hoc* Python scripts and visualized using Prism 9 software. The logo plot in Figure 2C was performed as previously described (Barber et al., 2021) using Logomaker (Tareen and Kinney, 2020).

dCas9-antibody fusion experiments

Plasmids encoding ATC-inducible dCas9 (pdCas9-bacteria #44249) and constitutively expressed gRNA (pgRNA-bacteria #44251) were obtained from Addgene (Qi et al., 2013). pdCas9-bacteria was modified to contain a C-terminal fusion of a nanobody that binds a peptide from β-catenin (nanobody BC2-Nb; Addgene #186420) (Braun et al., 2016; Traenkle et al., 2015) or an scFv that binds the spike protein from SARS-CoV-2 (ultrapotent B1-182.1; Addgene #186421) (Wang et al., 2021), in addition to a 6His tag for purification. These plasmids were co-transformed with pgRNA-bacteria encoding gRNA spacers 5′-TCCATAGATTTCTCCGTGAG-3′ and 5′-TGTTAGTTGCCCCATATCTT-3′, respectively, into BL21 *E. coli*. Protein expression and purification was performed as above, using only ATC for induction. GST fused to the beta catenin peptide recognized by the nanobody was also expressed and purified in a similar manner in BL21 (plasmid Addgene #186422). Recombinant spike protein ectodomain with stabilizing mutations was purchased from Sino Biological (40589-V08H4).

Approximately 10 μg of recombinant GST-beta catenin peptide or 4 μg spike protein were added to wells of a 96-well MaxiSorp plate (ThermoFisher) at ambient temperature for 2 h, shaking at 40 rpm. Wells were washed 6 times with 100 μL PBST and then treated with 100 μL of 100 mg/mL BSA at 23°C for 1 h, shaking at 40 rpm. Wells were again washed with 6 times with 100 μL PBST. Mixtures of approximately 2 μg each dCas9-nanobody and dCas9-scFv complexed with their respective gRNA barcodes were then added to each well in a 20 μL final volume (diluted using storage buffer) and incubated at 4°C for 16 h. Wells were then washed with 12 × 100 μL PBST. 20 μL water was then added to each well, and the plate was heated to 100°C in an oven for 10 min to elute gRNAs. 11 μL eluate was then used at the template for reverse transcription using Super-Script IV (Thermo) and the manufacturer's recommended protocols using primer 5′- GCACCGACTCGGTGCCACTTTTTC-3′. gRNAs were then amplified using barcode-specific primers (i.e. one primer anneals within the gRNA spacer region: 5′-TCCATAGATTTCTCCGTGAG-3′ or 5′-TGTTAGTTGCCCCATATCTTG-3′, with common reverse primer 5′-GCACCGACTCGGTG-3′) using Q5 (NEB) with 63°C annealing temperature, 10 s extension, and 45–50 total amplification cycles. Amplicons were run on a 2% w/v agarose gel and visualized with UV light. Amplicon band intensities were measured using ImageJ.

Microarray-based experiments using dCas9-scFv fusion B1-182.1 were performed by adding approximately 1 μg of the purified dCas9-fusion (with gRNA spacer 5′-TCCATAGATTTCTCCGTGAG-3′) and a negative control dCas9-6His fusion (with gRNA spacer 5′-CCGTACCTAGATACACTCAA-3′) to a double stranded DNA microarray harboring triplicate complementary probe sequences. The microarray was incubated for 16 h at 37°C. The microarray was then blocked with 2% milk in PBST for 30 min at 23°C. Then, approximately 100 ng of purified recombinant SARS-CoV-2 spike protein (Sino Biological 40589-V08H4 was added to the microarray and incubated at 23°C for 1 h. After washing twice with 40 μL PBST, 1:100 anti-SARS-CoV-2 spike CR3022 human IgG antibody (Cell Signaling 37475) was added to the microarray and incubated at 23°C for 1 h. The microarray was then washed twice with PBST and then incubated with 1:40 Alexa 647-conjugated anti-human IgG Fc antibody (Biolegend 410714) at 23°C for 1 h. The microarray was then visualized using a Genepix 4300A microarray scanner, and fluorescence intensities were extracted and analyzed as previously described (Barber et al., 2021; Shrock et al., 2020; Xu et al., 2015; Mina et al., 2019).

QUANTIFICATION AND STATISTICAL ANALYSIS

All bar graphs were produced in Prism 9. All sample sizes for relevant experiments are stated in figure legends. The nature of replicates is stated in each figure legend. All error bars are SD centered at the mean.

Cell Reports Methods, Volume *2*

Supplemental information

CasPlay provides a gRNA-barcoded CRISPR-based display platform for antibody repertoire profiling

Karl W. Barber, Ellen Shrock, and Stephen J. Elledge

Supplementary Fig. 1: Detailed CasPlay plasmid library cloning overview, related to STAR Methods

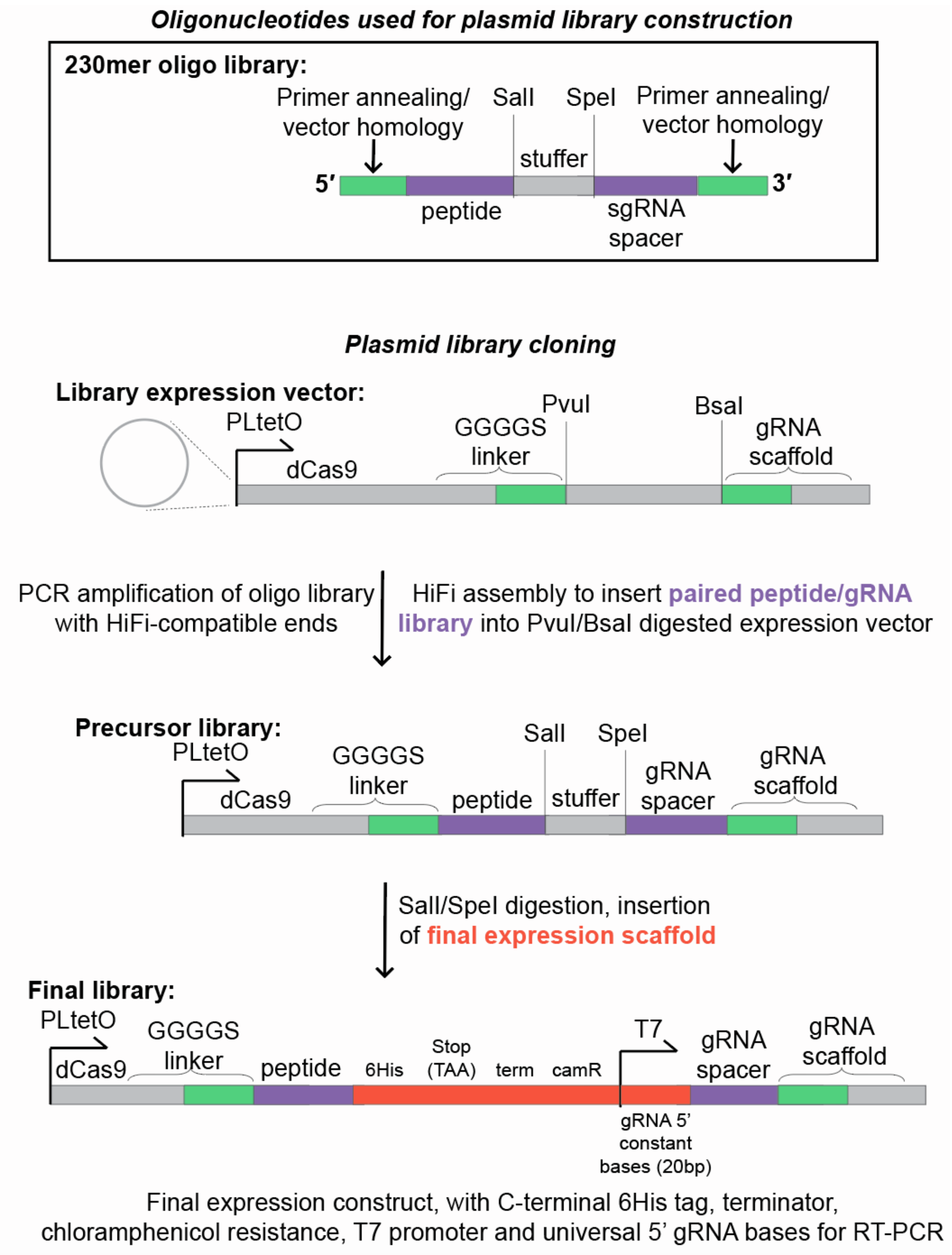

Schematic overview of CasPlay oligonucleotide library DNA sequence features and library cloning methods.

Supplementary Fig. 2: Cross-platform comparison of SARS-CoV-2 epitope mapping, related to Fig. 3

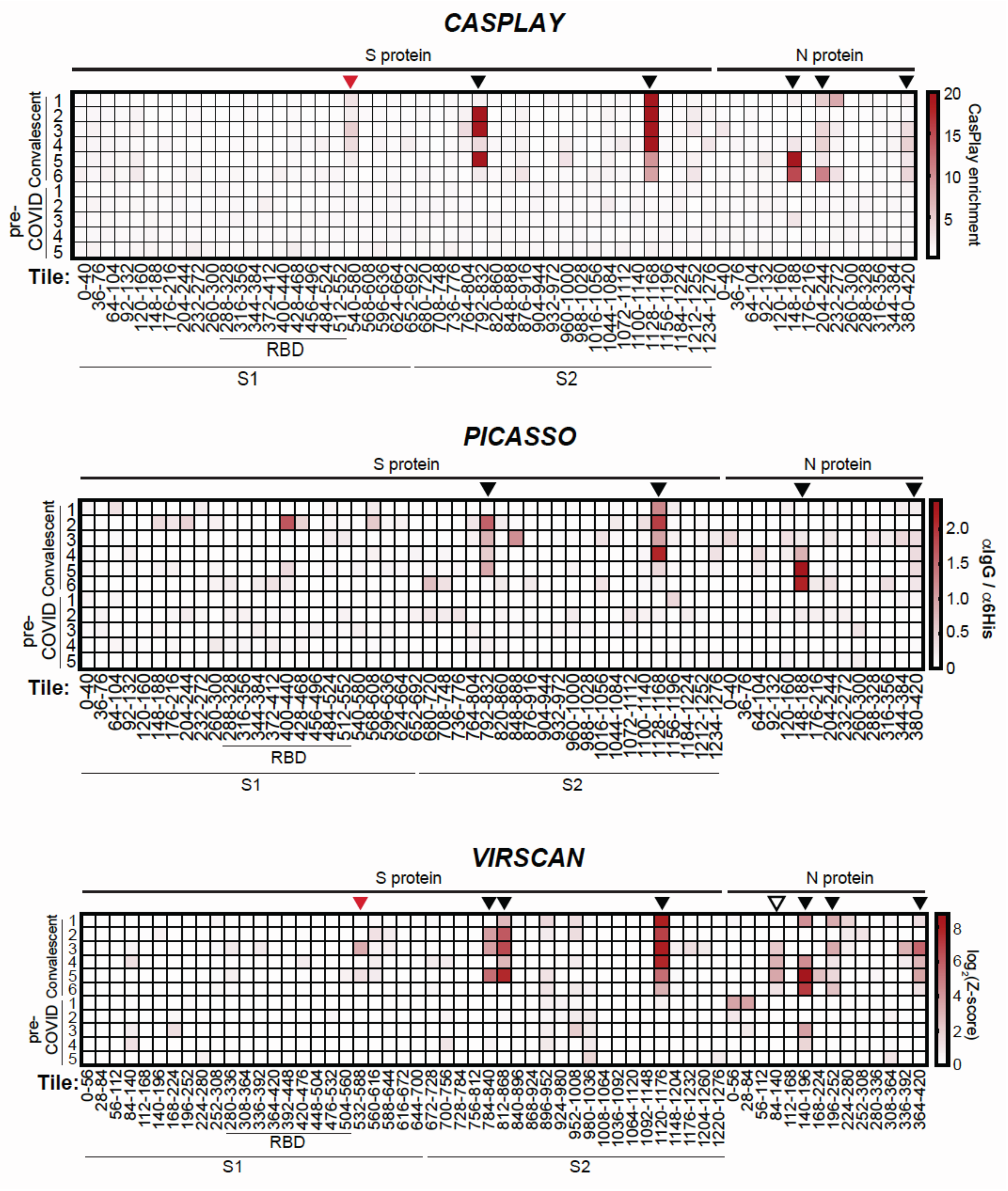

Matched patient sample data (6 convalescent patient serum samples, 5 pre-COVID serum samples) using tiled peptides representing SARS-CoV-2 shown. PICASSO and VirScan data obtained in and adapted from Barber et al., Molecular Cell 2021(Barber et al., 2021). Black arrowheads indicate epitopes recognized by two or more patients in this dataset above signal thresholds (CasPlay enrichment ≥ 3.5, PICASSO signal ratio ≥ 1.3, VirScan z-score ≥ 3.5). White arrowhead indicates epitope identified only by VirScan. Red arrowhead indicates epitope identified in both CasPlay and VirScan but not PICASSO, but only in one patient in this set.

Supplementary Fig. 3: CasPlay human virome library characterization and control experiments, related to Fig. 4

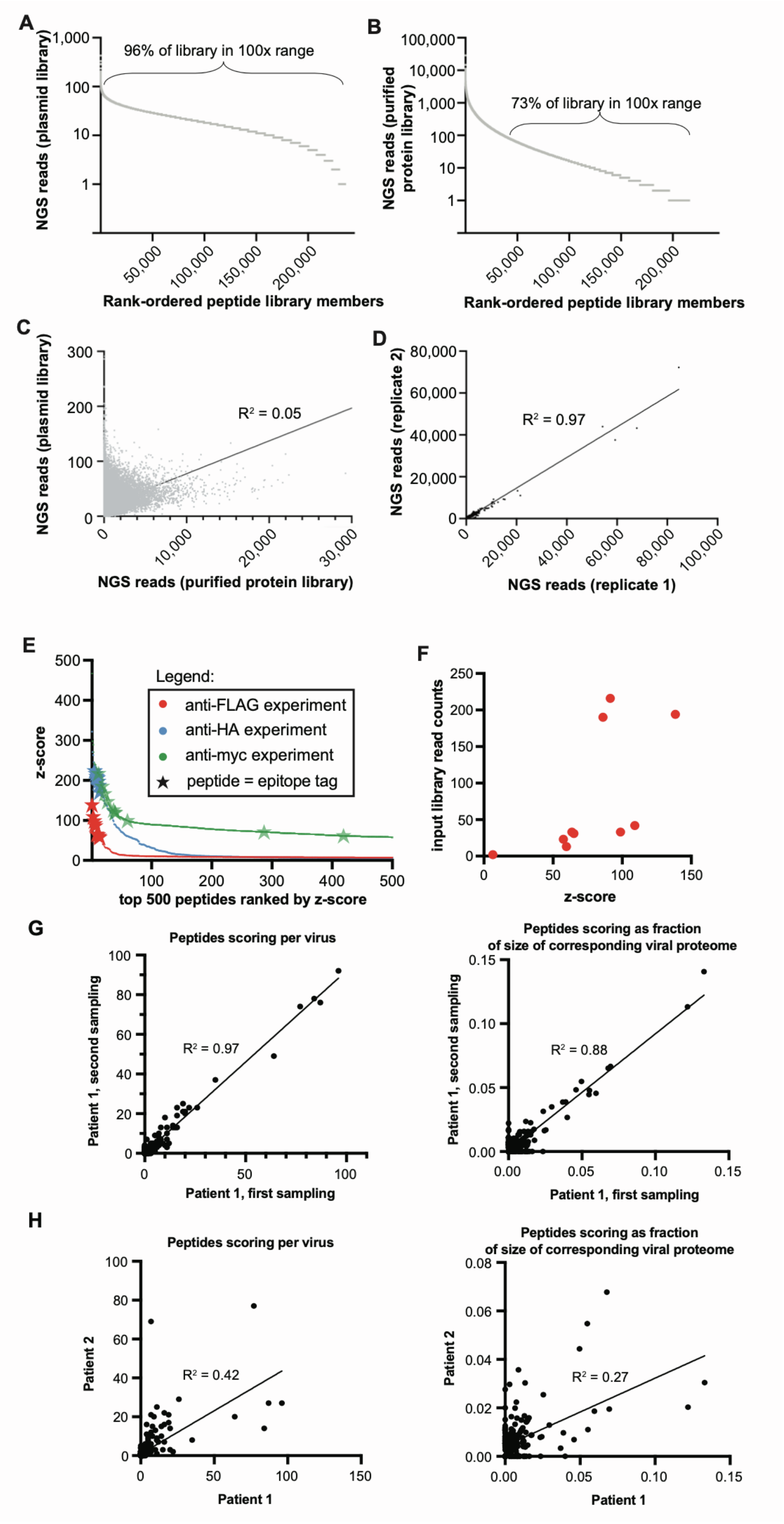

(**A**) Sequencing read distribution of 245,002 barcodes in final plasmid library encoding dCas9-peptide fusion and gRNA pairs. (**B**) Sequencing read distribution of 245,002 barcodes from RT-PCR of gRNAs from final purified dCas9-fusion library preparations. (**C**) No correlation between gRNA barcode read counts in final plasmid library and purified protein library. Three data points did not fall into the plotted range. (**D**) High correlation between independent sample replicates (from two separate transformations of the plasmid library) of gRNA barcodes sequenced from purified protein library. (**E**) Top 500 enriched peptides from the 245,002-member CasPlay library experiments using anti-FLAG, anti-HA or anti-myc antibody ranked by z-score of corresponding gRNA barcode. Epitope tag replicates corresponding to the designated antibody indicated by star symbols. Peptide z-scores averaged for two independent sample replicates. All expected control peptides are plotted, with the exception of one FLAG peptide which ranked #525 in the anti-FLAG immunoprecipitation. (**F**) Comparison of CasPlay z-scores for the FLAG control peptides in the anti-FLAG immunoprecipitation experiment and the barcode read counts in the 245,002-member CasPlay input library, averaged for two independent sample replicates. (**G**) Example of stability of measured antibody repertoire by CasPlay demonstrated by number of peptide hits (z-score ≥ 3.5, left) or peptide hits as a fraction of the size of a given viral proteome (right) in the CasPlay library from two patient-matched longitudinal samples, taken between two weeks and three months apart. (**H**) Example of inconsistency of peptide hits as total peptide hits (z-score ≥ 3.5, left) or peptide hits as fraction of size of corresponding viral proteome (right) for two unrelated samples.

Supplementary Fig. 4: Comparison of CasPlay to VirScan, related to Fig. 4

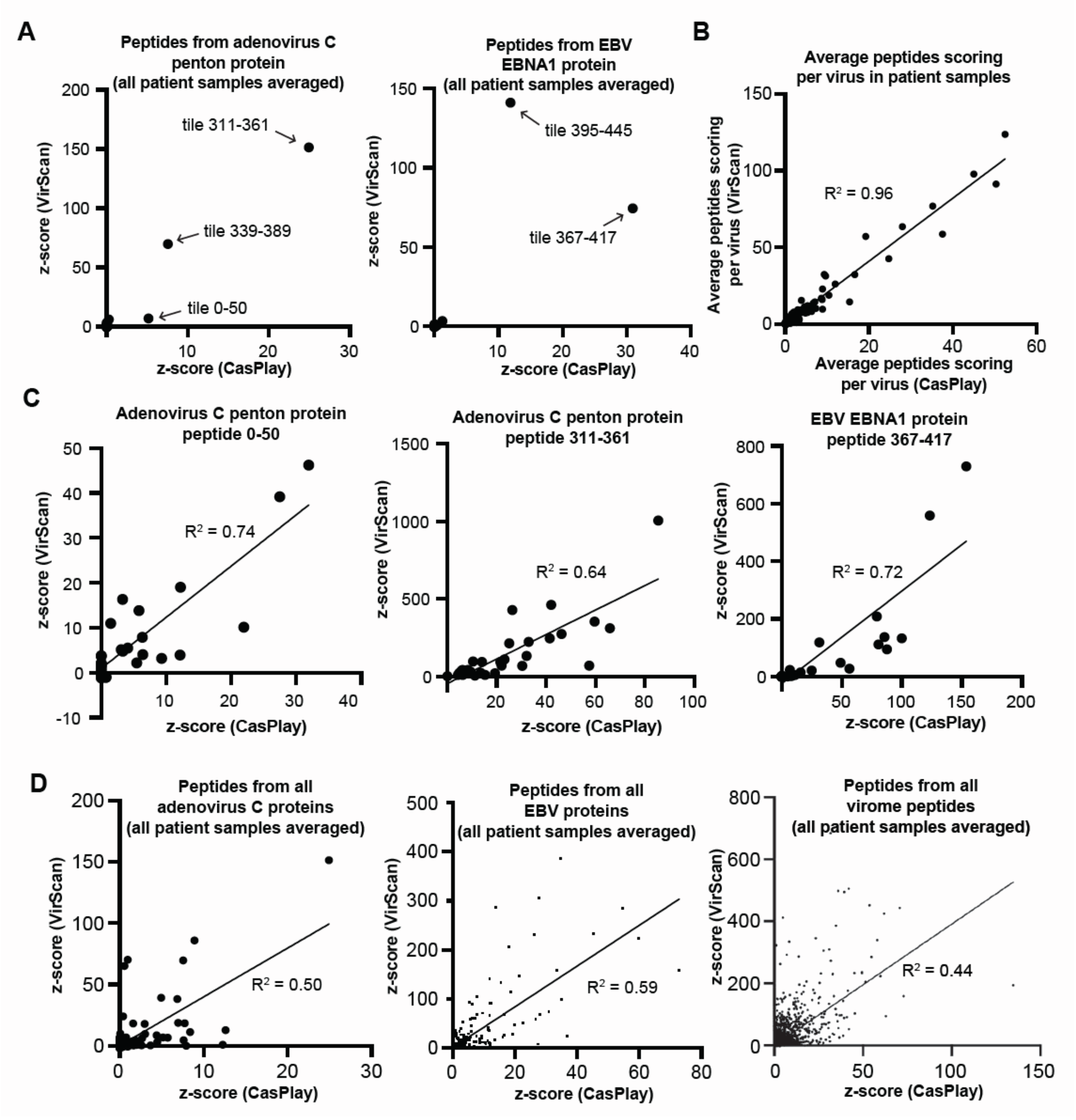

(**A**) Public epitopes from adenovirus C penton protein and EBV EBNA1 protein previously discovered by VirScan(Xu et al., 2015) were also uncovered by CasPlay (z-score ≥ 3.5). z-scores for each peptide from the annotated protein were averaged for 30 patient serum samples (with no suppositions about previous viral infection or vaccination status), measured in duplicate. (**B**) Average number of peptides scoring per

virus (for peptide z-score ≥ 3.5) across all 30 tested patient samples, compared between VirScan and CasPlay. (**C**) Correlation of peptide z-scores for representative public epitopes from 30 patient serum samples plotted individually, measured in duplicate. (**D**) Correlation of z-scores for each peptide derived from individual viruses or the entire virome averaged for 30 patient samples, compared between CasPlay and VirScan.

Supplementary Fig. 5: PICASSO-based application of dCas9-full length protein fusions, related to Fig. 5

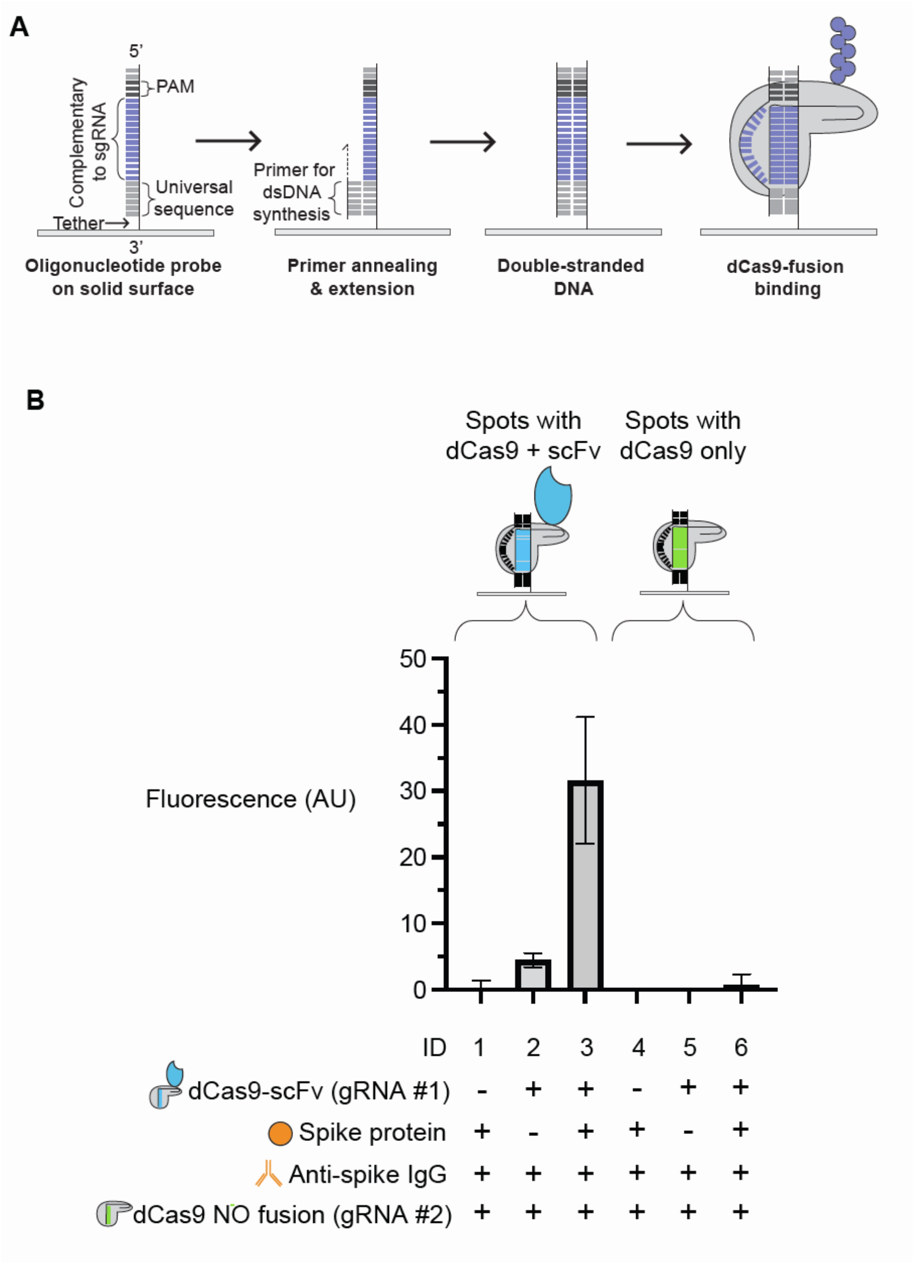

(**A**) Strategy for presenting a dCas9-fusion protein via PICASSO, adapted from Barber, et al., 2021(Barber et al., 2021). (**B**) dCas9-scFv fusion (anti-spike B1-182.1) presented via PICASSO enables fluorescence-based detection of corresponding analyte (spike protein). n = 6 replicate microarray features per condition.

Supplementary Table 1: Top 10 viruses with the most relative peptide hits by CasPlay and VirScan, related to Fig. 4

The average number of peptide hits (z-score ≥ 3.5) per virus per patient sample, reported as number of peptides or number of peptides as a percentage of the number of all peptides derived from that virus in the library by CasPlay and VirScan. Viruses are sorted by average peptide hits in CasPlay as a percentage of the viral proteome size, with viruses with fewer than 100 proteome peptides removed.

Virus	CasPlay - average peptide hits	VirScan - average peptide hits	Total peptides from virus encoded in library	CasPlay - peptide hits as percentage of encoded viral proteome	VirScan - peptide hits as percentage of encoded viral proteome
Human respiratory syncytial virus	52.5	123.7	781	6.7	15.8
Rhinovirus B	16.7	32.3	257	6.5	12.6
Rhinovirus A	35.2	76.9	660	5.3	11.7
Enterovirus B	45.1	97.8	1336	3.4	7.3
Enterovirus C	28.0	63.6	1009	2.8	6.3
Human parvovirus B19	4.3	8.9	169	2.6	5.3
Human herpesvirus 4	50.4	91.3	1980	2.5	4.6
Human adenovirus C	10.5	18.9	652	1.6	2.9
Human herpesvirus 1	24.8	42.7	1606	1.5	2.7
Influenza B virus	9.4	32.4	875	1.1	3.7
